# Attention-based workload prediction and dynamic resource allocation for heterogeneous computing environments

**DOI:** 10.1038/s41598-026-38622-4

**Published:** 2026-02-12

**Authors:** Shijia Shao, Xinyi Ding, Biao Zhao, Peiqing Ye

**Affiliations:** 1 School of Software, Northwestern Polytechnical University, Xi’an, 710129 Shaanxi China; 2 Shanghai Yingzhong Information Technology Co., LTD, Shanghai, 201600 China; 3 Shanghai Zhizhonglian Electronic Sales Co., Ltd., Shanghai, 201600 China; 4 School of Mechanical Engineering, Tsinghua University, Beijing, 100084 China

**Keywords:** Attention mechanism, Workload prediction, Resource allocation, Heterogeneous computing, Dynamic scheduling, AI workloads, Engineering, Mathematics and computing

## Abstract

The rapid proliferation of artificial intelligence applications in modern data centers demands intelligent resource management strategies that can effectively handle diverse workloads across heterogeneous computing infrastructures. This paper proposes an integrated framework that combines multi-head spatial-temporal attention mechanisms for workload prediction with dynamic resource allocation algorithms optimized for heterogeneous environments. The spatial-temporal attention architecture separately models temporal evolution patterns within individual workload streams and spatial correlations across concurrent task types, enabling accurate forecasting of resource demands. The allocation framework formulates resource assignment as a multi-objective optimization problem that jointly considers performance, energy efficiency, and utilization while explicitly accounting for prediction uncertainty. Experimental evaluation on real-world cluster traces demonstrates that our approach achieves 78.4% resource utilization with only 2.3% SLA violations, reduces average task completion time by 25.8%, and decreases energy consumption by 15.1% compared to production-grade baseline methods. The framework provides practical benefits for cloud service providers and enterprise data centers seeking to maximize infrastructure efficiency while maintaining service quality guarantees.

**Supplementary Information:**

The online version contains supplementary material available at 10.1038/s41598-026-38622-4.

## Introduction

The explosive growth of artificial intelligence applications has fundamentally reshaped the computational landscape of modern data centers, where diverse workloads ranging from deep learning training to real-time inference demand unprecedented levels of resource orchestration^1^. Cloud service providers now face mounting pressure to maximize resource utilization while maintaining quality-of-service guarantees across increasingly heterogeneous computing infrastructures that integrate CPUs, GPUs, FPGAs, and specialized AI accelerators^2^. This complexity intensifies as organizations deploy multiple AI frameworks and model architectures simultaneously, each exhibiting distinct computational patterns and resource consumption behaviors that traditional static allocation schemes struggle to accommodate effectively.

Current approaches to workload prediction and resource allocation reveal significant limitations when confronted with the dynamic nature of AI workloads. Conventional methods often rely on historical statistical analysis or simple time-series forecasting, which inadequately capture the intricate temporal dependencies and burst patterns characteristic of modern neural network training and inference tasks^3^. Meanwhile, existing resource allocation strategies typically operate on predetermined policies that fail to adapt to rapidly changing workload compositions, resulting in either resource wastage during low-demand periods or performance degradation when demand surges unexpectedly^4^. The mismatch between predicted and actual resource requirements frequently leads to service-level agreement violations, particularly for latency-sensitive inference workloads where millisecond-level delays can cascade into significant user experience degradation.

Research efforts in both academia and industry have explored various directions to address these challenges. Machine learning-based prediction models have shown promise in capturing workload patterns, yet many existing approaches treat prediction and allocation as separate optimization problems rather than unified processes^5^. Several studies have investigated reinforcement learning techniques for dynamic resource management, though convergence speed and sample efficiency remain problematic in production environments where exploration costs translate directly to service disruptions^6^. Recent work has begun incorporating attention mechanisms into workload characterization, recognizing that different temporal features contribute variably to prediction accuracy depending on workload phases and system states^7^. However, these efforts have not fully exploited the potential of attention-based architectures to jointly optimize prediction accuracy and allocation efficiency across heterogeneous computing resources.

The pressing need for more sophisticated solutions stems from several converging factors. First, the economic imperative to reduce operational costs in large-scale data centers demands intelligent resource management that minimizes both under-utilization and over-provisioning. Second, the environmental impact of computational infrastructure requires energy-efficient scheduling strategies that align workload placement with hardware capabilities. Third, the proliferation of edge computing scenarios introduces new constraints where resource scarcity and distributed coordination compound the allocation challenge. Addressing these multifaceted requirements necessitates a holistic framework that seamlessly integrates predictive analytics with adaptive resource orchestration.

This paper proposes an attention mechanism-based approach that fundamentally rethinks the coupling between workload prediction and resource allocation for AI workloads in heterogeneous computing environments^8^. Our primary contributions include: (1) a multi-head attention architecture that dynamically weighs temporal and spatial features for improved workload forecasting accuracy; (2) a joint optimization framework that considers prediction uncertainty when making allocation decisions, thereby reducing the impact of forecast errors on system performance; (3) a heterogeneity-aware scheduling algorithm that matches workload characteristics with specific accelerator capabilities to maximize throughput and minimize energy consumption. Rather than treating prediction and allocation as sequential stages, we develop an end-to-end learnable system where allocation feedback continuously refines prediction models, creating a closed-loop improvement cycle that adapts to evolving workload patterns without manual intervention.

## Related theory and technical foundation

### AI workload characteristic analysis and prediction technology

AI workloads exhibit distinctive temporal patterns that fundamentally differ from traditional enterprise applications, presenting unique challenges for prediction systems. Training workloads for deep neural networks often demonstrate strong temporal autocorrelation, where resource consumption at time $t$ closely relates to consumption at previous time steps through the relationship ${W}_{t}=f({W}_{t-1},{W}_{t-2},...,{W}_{t-n})$, though the depth of this dependency varies considerably across different model architectures^9^. Inference workloads, by contrast, display pronounced burstiness driven by user request patterns and upstream service behaviors, creating sudden spikes that can exceed baseline resource demands by orders of magnitude within seconds^10^. These burst characteristics pose particular difficulty because the magnitude and duration of spikes rarely follow predictable distributions that conventional smoothing techniques can adequately capture.

Periodicity emerges as another salient feature, particularly in production environments where training jobs often execute on scheduled intervals while inference traffic mirrors human activity cycles across daily, weekly, and sometimes seasonal timeframes. The superposition of multiple periodicities complicates prediction, as a single workload stream may simultaneously exhibit 24-h diurnal patterns, 7-day weekly rhythms, and longer-term trends that standard Fourier decomposition struggles to disentangle cleanly^11^.

Traditional time series forecasting approaches, including ARIMA models and exponential smoothing variants, attempt to model workload evolution through statistical representations of historical data. The ARIMA framework expresses future values through autoregressive and moving average components as $\varphi\:\left(B\right)(1-B{)}^{d}{W}_{t}=\theta\:(B){\epsilon}_{t}$ (standard ARIMA formulation), where $B$ denotes the backshift operator and ${\epsilon}_{t}$ represents white noise. Yet these methods presume stationarity and linear dependencies, assumptions that AI workloads routinely violate when training phases shift or when model serving patterns change abruptly^12^.

Machine learning approaches brought greater flexibility through algorithms like support vector regression and random forests, which detect nonlinear relationships between features and future resource demands^13^. Their main weakness lies in manual feature engineering requirements and limited capacity to capture long-range temporal dependencies spanning hundreds or thousands of time steps. Deep learning methods, particularly recurrent neural networks and their variants, address this limitation by automatically learning hierarchical representations from raw time series data^14^. The hidden state update mechanism ${h}_{t}=\mathrm{t}\mathrm{a}\mathrm{n}\mathrm{h}({W}_{hh}{h}_{t-1}+{W}_{xh}{x}_{t}+{b}_{h})$ (standard RNN formulation) enables information propagation across time, though vanilla RNNs suffer from vanishing gradients that constrain their effective memory horizon^15^.

Evaluating prediction quality requires metrics beyond simple error measures. Mean Absolute Percentage Error quantifies relative prediction accuracy, while coverage probability assesses whether prediction intervals contain actual values at specified confidence levels. For resource allocation decisions, we need metrics that account for asymmetric costs—underestimation triggers performance degradation while overestimation wastes resources, and these consequences carry different operational weights that prediction systems must balance thoughtfully.

Despite these advances, existing prediction approaches exhibit several critical limitations that our work specifically addresses. Traditional statistical methods like ARIMA assume stationarity and struggle with the non-linear, multi-modal distributions common in AI workloads. Deep learning approaches, while more flexible, typically model each workload stream independently and fail to capture cross-task correlations that arise from shared infrastructure resources. Recent transformer-based methods have shown promise; for instance, the variational mode decomposition combined with sample entropy optimization and Transformer architecture demonstrated improved cloud resource load prediction by decomposing complex signals into intrinsic mode functions^16^. However, such decomposition-based approaches introduce additional computational overhead and may lose fine-grained temporal dependencies during the signal separation process. Our spatial-temporal attention mechanism offers a fundamentally different approach: rather than pre-processing signals through decomposition, we learn to attend directly to relevant temporal and spatial patterns end-to-end, preserving information flow while reducing pipeline complexity. This distinction becomes particularly important for real-time scheduling decisions where prediction latency directly impacts allocation quality.

### Attention mechanism principles and applications

Self-attention mechanisms have revolutionized sequence modeling by enabling models to weigh the relevance of different positions when processing each element, essentially allowing the network to decide which parts of the input deserve more focus^17^. The fundamental operation transforms input sequences into query, key, and value representations through learned projections, then computes attention scores that measure the affinity between queries and keys. This scoring mechanism follows the formulation:

1 $${\mathrm{Attention}}(Q,K,V) = {\mathrm{softmax}}\left( {\frac{{QK^{T} }}{{\sqrt[{}]{{d_{k} }}}}} \right)V$$

where $Q$, $K$, and $V$ represent query, key, and value matrices respectively, while ${d}_{k}$ denotes the dimensionality of key vectors^18^. The scaling factor $\sqrt[]{{d}_{k}}$ prevents the dot products from growing excessively large, which would push the softmax function into regions with vanishingly small gradients during training.

Multi-head attention extends this concept by computing attention in parallel across multiple representation subspaces, capturing diverse dependency patterns that a single attention function might miss^19^. Each head performs independent attention operations with its own learned projection matrices, and the mechanism concatenates their outputs before applying a final linear transformation:

2 $${\mathrm{MultiHead}}(Q,K,V) = {\mathrm{Concat}}({\mathrm{head}}_{1} ,...,{\mathrm{head}}_{h} )W^{O}$$

where ${\mathrm{h}\mathrm{e}\mathrm{a}\mathrm{d}}_{i}=\mathrm{A}\mathrm{t}\mathrm{t}\mathrm{e}\mathrm{n}\mathrm{t}\mathrm{i}\mathrm{o}\mathrm{n}(Q{W}_{i}^{Q},K{W}_{i}^{K},V{W}_{i}^{V})$ (standard multi-head attention formulation) and ${W}^{O}$ denotes the output projection matrix^20^. This parallel processing enables the model to jointly attend to information from different positions and representation subspaces, something sequential models accomplish only through multiple stacked layers.

Spatial-temporal attention architectures specifically address scenarios where data exhibits both temporal dynamics and spatial correlations, a common situation in distributed computing environments where multiple servers or accelerators interact^21^. These designs typically separate temporal and spatial attention modules or employ factorized attention patterns that first capture dependencies within temporal dimensions before modeling spatial relationships, thereby reducing computational complexity from quadratic to linear with respect to sequence length.

For time series prediction tasks, attention mechanisms offer several compelling advantages over recurrent architectures. They eliminate the sequential computation bottleneck that prevents efficient parallelization during training, dramatically reducing the time required to process long historical sequences^22^. More importantly, attention scores provide direct paths between any two positions in the sequence, circumventing the information bottleneck that forces RNNs to compress all history into fixed-size hidden states. The attention weight matrix itself becomes interpretable, revealing which historical time steps the model considers most relevant for predicting each future point^23^.

The computation of attention weights involves applying the softmax function to scaled dot-product similarities, yielding a probability distribution over input positions:

3 $$\alpha _{{ij}} = \frac{{{\mathrm{exp}}\left( {e_{{ij}} } \right)}}{{\sum\nolimits_{{k = 1}}^{n} {{\mathrm{exp}}\left( {e_{{ik}} } \right)} }}$$

where $e_{{ij}} = \frac{{q_{i} \cdot k_{j} }}{{\sqrt {d_{k} } }}$ (standard attention score formulation) quantifies the compatibility between query $i$ and key $j$^24^. This normalization ensures weights sum to one while amplifying differences between high and low relevance positions. The resulting weighted combination of values creates contextualized representations that embed information from across the entire input sequence, with contribution magnitudes determined by learned relevance rather than fixed positional proximity.

### Heterogeneous computing resource scheduling strategies

Modern data centers embrace architectural diversity, deploying CPUs, GPUs, TPUs, and FPGAs within unified infrastructures to match workload characteristics with hardware strengths. CPUs excel at sequential logic and branching operations but deliver limited parallelism for matrix-intensive computations that dominate neural network training^25^. GPUs provide thousands of lightweight cores optimized for throughput-oriented tasks, achieving 10-100x speedups over CPUs for dense linear algebra operations, though their memory hierarchies and programming models introduce complexities that not all workloads can exploit efficiently^26^. TPUs represent domain-specific accelerators designed explicitly for tensor operations, offering superior performance per watt for transformer models and convolutional networks, yet their specialized instruction sets constrain flexibility when executing non-standard computational graphs. This diversity creates both opportunity and complexity—schedulers must navigate a multidimensional design space where placement decisions profoundly impact performance, cost, and energy consumption.

Static allocation strategies partition resources among workloads according to predetermined policies that remain fixed throughout execution. These approaches often assign resources based on historical average demands or worst-case requirements, guaranteeing each workload receives sufficient capacity but frequently leaving resources idle during low-utilization periods^27^. The simplicity of static schemes appeals to administrators seeking predictable behavior, yet their inflexibility proves costly when workload patterns shift or when bursty applications create temporal imbalances across the resource pool.

Dynamic allocation strategies continuously adjust resource assignments in response to runtime conditions, monitoring current utilization and redistributing capacity to match evolving demands^28^. Reactive policies increase allocations when detecting resource pressure while reclaiming underused resources for reassignment, though determining appropriate thresholds and response timescales poses challenges. Predictive dynamic schedulers attempt to anticipate future needs, preemptively adjusting allocations before demand materializes, but their effectiveness hinges critically on prediction accuracy^29^.

Heuristic scheduling algorithms apply domain knowledge and approximate optimization techniques to navigate the combinatorial complexity of resource assignment problems. Genetic algorithms encode allocation decisions as chromosomes and evolve populations toward improved solutions through selection and mutation operators, though convergence speed remains problematic for online scheduling scenarios^30^. Greedy heuristics make locally optimal choices at each decision point, offering computational efficiency at the cost of potentially missing globally superior configurations.

Resource utilization quantifies the fraction of available capacity actively performing useful work, formulated as:

4 $$U = \frac{1}{T}\sum\limits_{{t = 1}}^{T} {\frac{{\sum\nolimits_{{i = 1}}^{N} {r_{i} \left( t \right)} }}{{R_{{total}} }}}$$

where ${r}_{i}\left(t\right)$ represents resource consumption of workload $i$ at time $t$ and ${R}_{total}$ denotes total capacity (standard utilization definition). High utilization indicates efficient resource employment yet risks performance degradation when demand spikes exceed available headroom.

Minimizing task completion time directly impacts user experience and throughput, while energy consumption increasingly dominates operational expenses and environmental considerations. Multi-objective optimization frameworks balance these competing goals through weighted combinations or Pareto optimality:

5 $${\mathrm{min}}_{x} \left[ {\alpha \cdot T_{{completion}} \left( x \right) + \beta \cdot E_{{total}} \left( x \right) - \gamma \cdot U\left( x \right)} \right]$$

where $x$ represents the allocation decision vector and $\alpha\:,\beta\:,\gamma$ denote preference weights (standard multi-objective formulation)^31^. Determining appropriate weight configurations requires understanding application-specific priorities and system constraints that vary across deployment contexts.

Beyond performance and energy optimization, recent research has increasingly recognized the importance of reliability, fault-tolerance, and security in heterogeneous computing environments. RT-SEAT introduced a hybrid real-time scheduling approach that jointly optimizes energy consumption and thermal management for heterogeneous multicore platforms, demonstrating that thermal-aware scheduling can prevent performance degradation from throttling^32^. The HEAT scheduler extended this work by incorporating efficient temperature management mechanisms that proactively migrate tasks before thermal violations occur^33^. For mission-critical applications, FRESH proposed fault-tolerant scheduling strategies that maintain real-time guarantees even under processor failures, achieving graceful degradation rather than catastrophic system failures^34^. More recently, TREAFET addressed temperature-aware scheduling specifically for FinFET-based multicores, accounting for the unique thermal characteristics of advanced semiconductor processes^35^. Security considerations have also gained prominence, with SAMIT proposing secure multi-authority access control with dynamic attribute updates for IoT-CPS systems^36^, while e-SAFE addressed secure access control with user revocation capabilities in fog-enhanced IoT environments^37^. Our current framework focuses primarily on performance and energy optimization, deliberately leaving security and fault-tolerance mechanisms as explicit future extensions. We acknowledge this scope limitation while noting that the modular architecture we propose can accommodate such extensions without fundamental redesign.

## Attention mechanism-based workload prediction and resource allocation algorithm design

### Multi-head spatial-temporal attention-based workload prediction model

We construct a multi-head spatial-temporal attention network that decomposes the workload prediction problem into two complementary perspectives: temporal evolution within individual workload streams and spatial correlations across concurrent task types. This factorization reduces computational complexity while preserving the model’s capacity to capture intricate dependencies that simpler architectures miss^38^.

The temporal attention module processes historical workload sequences to identify which past time steps most strongly influence future resource demands. Given an input sequence $X\in\:{\mathbb{R}}^{T\times\:D}$ where $T$ represents the temporal window length and $D$ denotes feature dimensions, we project each time step into query, key, and value representations through learned linear transformations. The temporal attention computation follows:

6 $${A}_{temp}=\mathrm{s}\mathrm{o}\mathrm{f}\mathrm{t}\mathrm{m}\mathrm{a}\mathrm{x}\left(\frac{{Q}_{temp}{K}_{temp}^{T}}{\sqrt[]{{d}_{k}}}\right){V}_{temp}$$

where subscript “temp” indicates temporal-specific projection matrices that differ from those employed in spatial attention. This separation allows the model to learn distinct relevance patterns for temporal versus spatial relationships^39^. We implement multi-head temporal attention by partitioning the $D$ dimensions into $h$ heads, each operating on ${d}_{k}=D/h$ dimensions, enabling parallel capture of diverse temporal patterns such as short-term fluctuations and long-term trends simultaneously.

Spatial attention operates orthogonally to temporal attention, examining relationships between different workload types at each time step. In heterogeneous environments where training jobs, batch inference, and online serving coexist, resource contention and shared infrastructure create dependencies that naive per-workload predictions overlook. The spatial attention mechanism computes cross-workload affinities:

7 $${A}_{spatial}=\mathrm{s}\mathrm{o}\mathrm{f}\mathrm{t}\mathrm{m}\mathrm{a}\mathrm{x}\left(\frac{{Q}_{spatial}{K}_{spatial}^{T}}{\sqrt[]{{d}_{k}}}\right){V}_{spatial}$$

By attending across the workload dimension rather than the temporal dimension, this module identifies scenarios where one task type’s behavior signals impending changes in another—for instance, when training job completions typically precede surges in inference requests as newly trained models deploy^40^.

To ensure reproducibility, we now formally specify the computational process for both attention modules. For temporal attention, given input tensor $X\in\:{\mathbb{R}}^{B\times\:T\times\:N\times\:D}$ where $B$ denotes batch size, $T$ represents temporal window length, $N$ indicates the number of concurrent workload types, and $D$ specifies feature dimensions, we first reshape the tensor to ${X}_{temp}\in\:{\mathbb{R}}^{\left(B\cdot\:N\right)\times\:T\times\:D}$, treating each workload stream independently. The query, key, and value matrices are constructed through learned linear projections:

$${Q}_{temp}={X}_{temp}{W}_{temp}^{Q},\:{K}_{temp}={X}_{temp}{W}_{temp}^{K},\:{V}_{temp}={X}_{temp}{W}_{temp}^{V}$$

where ${W}_{temp}^{Q},{W}_{temp}^{K},{W}_{temp}^{V}\in\:{\mathbb{R}}^{D\times\:{d}_{k}}$ are learnable parameter matrices with ${d}_{k}=D/h$ for $h$ attention heads. We apply causal masking to prevent information leakage from future time steps:

$$M_{{causal}} \left( {i,j} \right) = \left\{ {\begin{array}{*{20}l} 0 \hfill & {{\mathrm{if}}\:{\mathrm{i}} \ge \:{\mathrm{j}}} \hfill \\ { - \infty } \hfill & {{\mathrm{otherwise}}} \hfill \\ \end{array} } \right.$$

The masked attention scores undergo softmax normalization row-wise, ensuring each query position attends only to current and preceding positions. For spatial attention operating across workload types, we reshape the temporally-attended output to ${X}_{spatial}\in\:{\mathbb{R}}^{\left(B\cdot\:T\right)\times\:N\times\:D}$ and apply analogous projections without causal masking, as all concurrent workloads can legitimately influence each other. The integration mechanism concatenates outputs from all heads and applies a final linear transformation followed by layer normalization and residual connection:

$${H}_{out}=\mathrm{LayerNorm}\left(X+\mathrm{Concat}\left(hea{d}_{1},...,hea{d}_{h}\right){W}^{O}\right)$$

This formulation ensures that temporal dependencies are captured before spatial correlations, allowing the model to first understand individual workload evolution patterns before reasoning about cross-workload interactions.

**Fig. 1:**
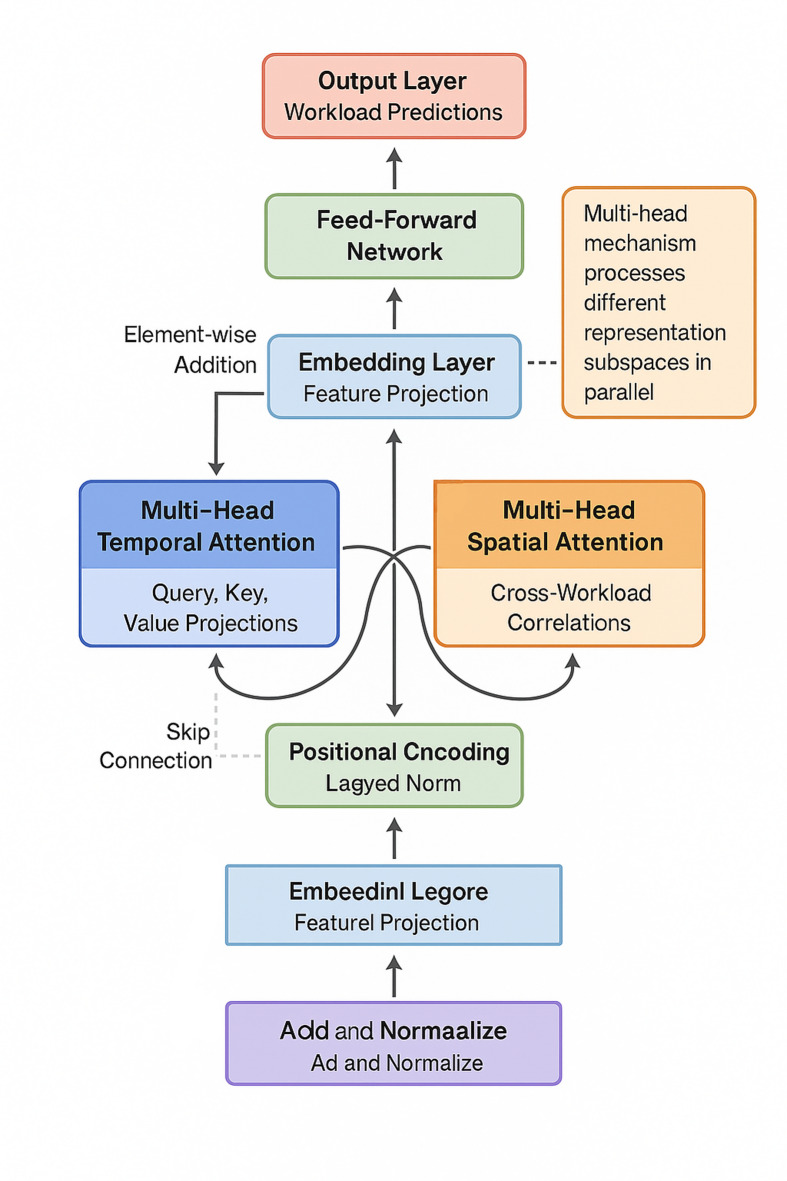
Multi-head spatial-temporal attention network architecture for workload prediction.

Figure 1 illustrates the complete network architecture, showing how temporal and spatial attention modules interleave to progressively refine representations. The input layer accepts normalized workload metrics including CPU utilization, memory consumption, network throughput, and GPU occupancy across multiple time steps and workload types.

Positional encoding injects temporal order information into the model, which attention mechanisms lack inherently due to their permutation-invariant nature. We adopt sinusoidal positional embeddings that encode absolute position through varying frequency components:

8 $$P{E}_{(pos,2i)}=\mathrm{s}\mathrm{i}\mathrm{n}\left(\frac{pos}{{10000}^{2i/D}}\right),P{E}_{(pos,2i+1)}=\mathrm{c}\mathrm{o}\mathrm{s}\left(\frac{pos}{{10000}^{2i/D}}\right)$$

where $pos$ denotes the position index and $i$ represents the dimension index (standard positional encoding formulation). These embeddings sum with input features before attention computation, enabling the model to distinguish temporal ordering without requiring recurrent connections.

Residual connections wrap each attention module, mitigating gradient degradation in deep networks while providing shortcuts that preserve low-level features. The formulation combines attention outputs with original inputs through element-wise addition:

9 $${H}^{(l+1)}=\mathrm{L}\mathrm{a}\mathrm{y}\mathrm{e}\mathrm{r}\mathrm{N}\mathrm{o}\mathrm{r}\mathrm{m}({H}^{\left(l\right)}+\mathrm{A}\mathrm{t}\mathrm{t}\mathrm{e}\mathrm{n}\mathrm{t}\mathrm{i}\mathrm{o}\mathrm{n}({H}^{\left(l\right)}\left)\right)$$

where ${H}^{\left(l\right)}$ represents the hidden state at layer $l$ and LayerNorm denotes layer normalization that stabilizes training dynamics^41^.

The end-to-end prediction pipeline processes raw workload time series through embedding layers, stacks multiple spatial-temporal attention blocks, and produces future workload forecasts via fully connected output layers. We train the model by minimizing the combined loss:

10 $${\mathcal{L}} = \frac{1}{N}\mathop \sum \limits_{{i = 1}}^{N} \left[ {(\hat{w}_{i} - w_{i} )^{2} + \lambda \cdot{\mathrm{max}}\left( {0,\hat{w}_{i} - c_{i} } \right)} \right]$$

where $\hat {{w}}_{i}$ and ${w}_{i}$ denote predicted and actual workloads, ${c}_{i}$ represents capacity constraints, and $\lambda$ penalizes predictions exceeding physical limits (custom loss formulation)^42^. This asymmetric penalty encourages the model to avoid generating infeasible predictions that would mislead downstream allocation algorithms.

Table 1 summarizes the key hyperparameters governing model architecture and training dynamics. As presented in Table 1, we configure 8 attention heads to balance representation capacity against computational overhead, while the embedding dimension of 512 provides sufficient expressiveness for capturing complex workload patterns without excessive parameter counts.

**Table 1:** Model parameter configuration for multi-head spatial-temporal attention network.

Parameter	Value	Parameter	Value	Rationale
Number of attention heads	8	Embedding dimension	512	Balances capacity and efficiency
Number of encoder layers	6	Dropout rate	0.1	Prevents overfitting
Temporal window length	128	Prediction horizon	32	Covers typical workload cycles
Feedforward dimension	2048	Learning rate	0.0001	Stable convergence
Batch size	64	Training epochs	200	Sufficient for convergence
Optimizer	Adam	Weight decay	0.01	Regularizes parameters
Warmup steps	4000	Gradient clipping	1.0	Prevents gradient explosion
Spatial attention heads	4	Temporal attention heads	4	Separate spatial/temporal focus

### Heterogeneous resource dynamic allocation algorithm framework

Building upon the workload predictions generated by our spatial-temporal attention model, we now establish a decision framework that translates forecasts into concrete resource assignments across heterogeneous computing infrastructure. The framework operates in three stages: resource state modeling, multi-objective allocation optimization, and adaptive adjustment through preemption and migration mechanisms when actual demands deviate from predictions.

Our resource pool modeling approach represents the heterogeneous infrastructure as a directed graph $\mathcal{G}=(\mathcal{N},\mathcal{E})$ where nodes $\mathcal{N}$ correspond to computing devices and edges $\mathcal{E}$ capture interconnection topology and bandwidth constraints^43^. Each node ${n}_{i}\in\:\mathcal{N}$ maintains a state vector ${s}_{i}\left(t\right)=\left[{c}_{i}\right(t),{m}_{i}(t),{g}_{i}(t),{e}_{i}(t){]}^{T}$ encoding available CPU cores, memory capacity, GPU resources, and current energy consumption rate respectively. Table 2 summarizes the key characteristics distinguishing different resource types within our heterogeneous pool. As presented in Table 2, GPUs deliver substantially higher throughput for parallel workloads but consume considerably more power per unit time compared to CPUs, creating inherent trade-offs that allocation algorithms must navigate carefully.

**Table 2:** Characteristics of heterogeneous computing resource types.

Resource type	Peak performance (TFLOPS)	Memory bandwidth (GB/s)	Power consumption (W)	Cost efficiency
CPU (32-core)	2.5	128	180	Moderate
GPU (V100)	14.0	900	300	High for parallel tasks
GPU (A100)	19.5	1555	400	Very high for AI workloads
TPU v4	275.0	1200	450	Optimal for transformers
FPGA	8.0	512	225	High for specific algorithms

The allocation decision at each time step determines a mapping $\varphi\::\mathcal{W}\to\:\mathcal{N}$ from workloads $\mathcal{W}$ to computing nodes, constrained by capacity limits and workload requirements^44^. We formulate this as a multi-objective optimization problem that balances performance, energy efficiency, and resource utilization:

11 $${\mathrm{min}}_{\phi } f\left( \phi \right) = \left[ {\begin{array}{*{20}l} {f_{1} \left( \phi \right) = \mathop \sum \limits_{{j \in {\mathcal{W}}}}^{{}} T_{j} \left( \phi \right){\mathrm{~}}f_{2} \left( \phi \right) = \mathop \sum \limits_{{i \in {\mathcal{N}}}}^{{}} E_{i} \left( \phi \right){\mathrm{~}}f_{3} \left( \phi \right) = - \frac{1}{{\left| {\mathcal{N}} \right|}}\mathop \sum \limits_{{i \in {\mathcal{N}}}}^{{}} U_{i} \left( \phi \right)} \\ \end{array} } \right]$$

where ${T}_{j}\left(\varphi\:\right)$ represents completion time for workload $j$, ${E}_{i}\left(\varphi\:\right)$ denotes energy consumed by node $i$, and ${U}_{i}\left(\varphi\:\right)$ measures utilization of node $i$ under allocation $\varphi$ (custom multi-objective formulation). The negative sign on the utilization term transforms maximization into minimization for consistent optimization direction.

Subject to constraints that ensure feasibility, allocations must respect resource capacities at all nodes:

12 $$\mathop \sum \limits_{{j:\phi \left( j \right) = i}}^{{}} r_{j}^{{cpu}} \le c_{i} \left( t \right),\mathop \sum \limits_{{j:\phi \left( j \right) = i}}^{{}} r_{j}^{{mem}} \le m_{i} \left( t \right),\forall i \in {\mathcal{N}}$$

where ${r}_{j}^{cpu}$ and ${r}_{j}^{mem}$ denote CPU and memory requirements of workload $j$ (standard capacity constraints). Additional constraints enforce workload-specific requirements, such as minimum GPU memory for training large language models or maximum network latency for real-time inference services^45^.

**Fig. 2:**
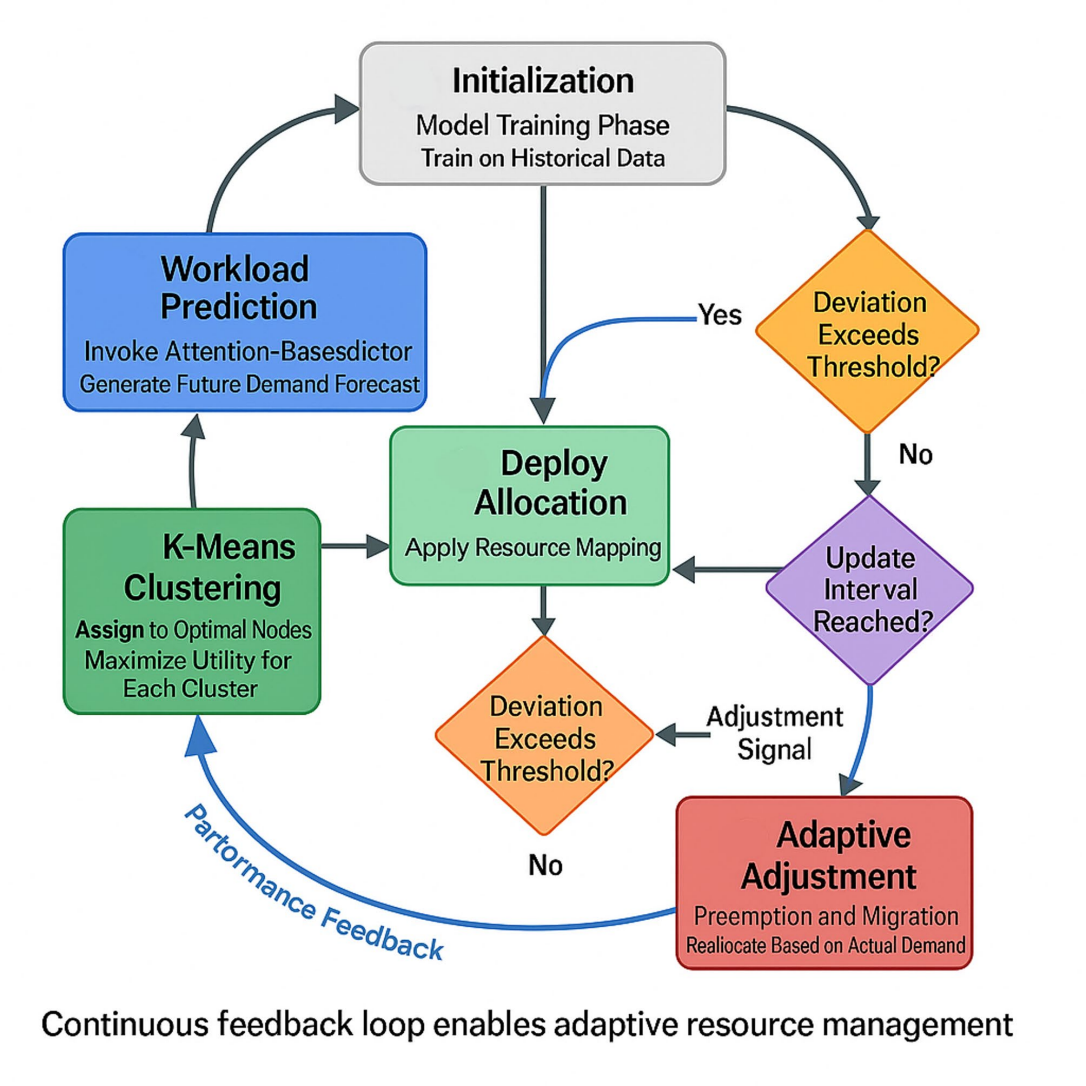
Dynamic resource allocation algorithm flowchart incorporating prediction and feedback.

Figure 2 demonstrates the complete allocation algorithm flow, beginning with workload prediction from the attention-based model and proceeding through initial allocation, runtime monitoring, and adaptive adjustments. The framework continuously compares actual resource consumption against predictions, triggering reallocation when deviations exceed predefined thresholds.

We employ a weighted Tchebycheff approach to scalarize the multi-objective problem, converting it into a single-objective formulation that explores the Pareto frontier systematically:

13 $${\mathrm{min}}_{\phi } {\mathrm{max}}_{{k = 1,2,3}} \left\{ {w_{k} \cdot\frac{{f_{k} \left( \phi \right) - f_{k}^{{\mathrm{*}}} }}{{f_{k}^{{worst}} - f_{k}^{{\mathrm{*}}} }}} \right\}$$

where ${w}_{k}$ represents user-specified weights reflecting objective priorities, ${f}_{k}^{\mathrm{*}}$ denotes the ideal value for objective $k$, and ${f}_{k}^{worst}$ represents the nadir point (standard Tchebycheff formulation)^46^. This approach generates diverse Pareto-optimal solutions by varying weight vectors, allowing administrators to select allocations matching their operational priorities.

Prediction errors inevitably occur despite sophisticated forecasting models, necessitating mechanisms that gracefully handle mismatches between anticipated and actual resource demands. Our preemption mechanism temporarily suspends lower-priority workloads when higher-priority tasks encounter resource shortages:

14 $${\mathrm{Preempt}}\left( j \right) \Leftrightarrow U_{i} \left( \phi \right) > \theta _{{high}} \wedge {\mathrm{Priority}}\left( j \right) < {\mathrm{min}}_{{k:\phi \left( k \right) = i}} {\mathrm{Priority}}\left( k \right)$$

where ${\theta}_{high}$ denotes the utilization threshold triggering preemption and Priority$\left(j\right)$ quantifies workload $j$‘s importance (custom preemption rule). Preempted workloads migrate to alternative nodes with available capacity or queue for future execution, preserving progress through checkpointing^47^.

The utilization threshold ${\theta}_{high}$ that triggers preemption is determined through an adaptive calibration procedure rather than static configuration. We initialize ${\theta}_{high}=0.85$ based on common industry practice, then adjust dynamically based on observed SLA violation rates over sliding windows. Specifically, if SLA violations exceed 5% over the past hour, we decrease ${\theta}_{high}$ by 0.02 to trigger earlier preemption; conversely, if violations remain below 1%, we increase ${\theta}_{high}$ by 0.01 to allow tighter resource packing. This adaptive mechanism converges to environment-specific thresholds that balance utilization against reliability. In our experiments, the threshold stabilized between 0.82 and 0.88 across different datasets, reflecting varying workload volatility characteristics. We also maintain a lower threshold ${\theta}_{low}=0.3$ below which nodes become candidates for consolidation and potential power-down during low-demand periods.

Migration decisions balance the cost of transferring workload state against the benefit of improved resource matching. We quantify this trade-off through explicit cost modeling:

$${\mathrm{Gain}}_{migrate}\left(j,i\to\:i{\prime\:}\right)=\varDelta\:{T}_{j}\cdot\:\mathrm{Priority}\left(j\right)-{C}_{transfer}\left(j,i\to\:i^{\prime\:}\right)$$

where $\varDelta\:{T}_{j}$ represents expected reduction in completion time and ${C}_{transfer}$ quantifies migration overhead^48^. The transfer cost decomposes into three measurable components:

$${C}_{transfer}\left(j,i\to\:i{\prime\:}\right)={t}_{checkpoint}+{t}_{network}+{t}_{warmup}$$

Here, ${t}_{checkpoint}={S}_{j}/{\mathrm{BW}}_{disk}$ represents checkpoint saving time proportional to workload state size ${S}_{j}$ and disk bandwidth; ${t}_{network}={S}_{j}/{\mathrm{BW}}_{net}\left(i,i{\prime\:}\right)$ captures network transfer time dependent on inter-node bandwidth; and ${t}_{warmup}$ accounts for cache warming and memory allocation delays on the destination node, empirically measured at 2–5 s for GPU workloads.

The resource matching improvement is computed as:

$$\varDelta\:{T}_{j}={T}_{j}^{current}\left(i\right)-{T}_{j}^{estimated}\left(i{\prime\:}\right)=\frac{{R}_{j}}{\mathrm{Perf}\left(i,{\mathrm{type}}_{j}\right)}-\frac{{R}_{j}}{\mathrm{Perf}\left(i^{\prime\:},{\mathrm{type}}_{j}\right)}$$

where ${R}_{j}$ denotes remaining computation and $\mathrm{Perf}\left(n,\mathrm{type}\right)$ represents the performance coefficient of node $n$ for workload type $\mathrm{type}$. This coefficient is pre-computed through microbenchmarking, capturing hardware-workload affinity (e.g., A100 GPUs achieve 1.7× performance coefficient for transformer training compared to V100s). Migrations proceed only when positive gain exceeds a minimum threshold of 30 s, preventing thrashing from marginal improvements.

The allocation algorithm iterates between prediction, optimization, and adjustment phases. Each cycle begins by invoking the attention-based prediction model to forecast workload demands over the upcoming planning horizon, typically spanning 15–30 min based on typical workload phase durations:

15 $$\hat {{W}}_{t+1:t+H}=\mathrm{P}\mathrm{r}\mathrm{e}\mathrm{d}\mathrm{i}\mathrm{c}\mathrm{t}\mathrm{o}\mathrm{r}({W}_{t-L:t},{S}_{t})$$

where $\hat {{W}}_{t+1:t+H}$ represents predicted workloads for horizon $H$, ${W}_{t-L:t}$ denotes historical workload observations over lookback window $L$, and ${S}_{t}$ captures current system state (prediction invocation). The optimization solver then determines initial allocations, which runtime monitors refine through preemption and migration as actual demands materialize.

### Algorithm optimization and implementation

Training the spatial-temporal attention prediction model requires careful design of both optimization strategy and loss function to ensure convergence toward accurate, robust forecasts. We adopt a curriculum learning approach that progressively increases prediction horizon length during training, beginning with single-step-ahead forecasts before advancing to longer horizons^49^. This strategy accelerates early learning by focusing initially on simpler prediction tasks, then gradually introducing the complexity of multi-step forecasting as model parameters stabilize. The training loss combines mean squared error with a quantile regression term that captures prediction uncertainty:

16 $${\mathcal{L}}_{{train}} = \frac{1}{N}\mathop \sum \limits_{{i = 1}}^{N} \left[ {(y_{i} - \hat{y}_{i} )^{2} + \mathop \sum \limits_{{q \in Q}}^{{}} \rho _{q} \left( {y_{i} - \hat{y}_{i}^{q} } \right)} \right]$$

where $\rho _{q} \left( e \right) = e\cdot\left( {q - 1_{{e < 0}} } \right)$ represents the quantile loss function at quantile level $q$, $\hat {{y}}_{i}^{q}$ denotes the $q$-th quantile prediction, and $Q=\left\{\mathrm{0.1,0.5,0.9}\right\}$ captures prediction intervals (quantile loss formulation). This formulation produces prediction distributions rather than point estimates, information that downstream allocation algorithms exploit when determining safety margins.

Solving the multi-objective resource allocation optimization problem exactly becomes computationally prohibitive as the number of workloads and nodes grows, motivating our development of an efficient heuristic that produces near-optimal solutions within tight time budgets. We decompose the allocation problem into two stages: first clustering workloads by resource profile similarity, then solving smaller subproblems for each cluster independently^50^. The clustering employs k-means on normalized resource requirement vectors, partitioning workloads into groups that likely benefit from similar accelerator types. Each subproblem then determines node assignments within its cluster through a greedy procedure that iteratively places workloads on nodes offering maximum utility gain:

17 $$n^{{\mathrm{*}}} = {\mathrm{argmax}}_{{n \in {\mathcal{N}}}} \left\{ {\Delta U\left( {j,n} \right) - \beta \cdot E_{n} \cdot\Delta t_{j} \left( n \right)} \right\}$$

where $\varDelta\:U(j,n)$ quantifies utilization improvement from assigning workload $j$ to node $n$, ${E}_{n}$ represents the energy consumption rate of node $n$, $\varDelta\:{t}_{j}\left(n\right)$ estimates execution duration, and $\beta$ balances performance against energy concerns (greedy selection rule). This decomposition reduces complexity from examining all possible assignments to considering only relevant subsets.

Workload characteristics drift over time as application mixes evolve and user behavior patterns shift, requiring the prediction model to adapt continuously rather than remaining static after initial training. Our online update mechanism implements incremental learning through periodic fine-tuning on recent data windows^51^. Every $\tau$ time steps, the system collects newly observed workload traces and performs gradient descent updates on model parameters:

18 $${\theta}_{t+\tau\:}={\theta}_{t}-\eta\:{\nabla}_{\theta\:}\mathcal{L}({\mathcal{D}}_{recent};{\theta}_{t})$$

where ${\theta}_{t}$ denotes model parameters at time $t$, $\eta$ represents a reduced learning rate for stability, and ${\mathcal{D}}_{recent}$ contains the most recent observations (online update rule). This approach maintains model relevance without discarding previously learned patterns, as the small learning rate prevents catastrophic forgetting of historical knowledge.

Computational complexity analysis reveals important scaling characteristics for practical deployment. The prediction model requires $O\left({T}^{2}\cdot\:D\cdot\:h+{N}^{2}\cdot\:D\cdot\:h\right)$ operations per forward pass, where the first term corresponds to temporal attention over sequence length $T$ and the second term captures spatial attention across $N$ concurrent workload types. For typical configurations ($T=128$, $N=50$, $D=512$, $h=8$), temporal attention dominates computation. The allocation algorithm exhibits $O\left(\left|\mathcal{W}\right|\cdot\:\left|\mathcal{N}\right|\cdot\:k\right)$ complexity after clustering, representing substantial improvement over exhaustive search scaling as $O\left({\left|\mathcal{N}\right|}^{\left|\mathcal{W}\right|}\right)$.

Table 3 presents empirical runtime measurements across varying cluster scales to validate these theoretical bounds. As cluster size increases from 20 to 500 nodes, prediction latency grows sub-linearly due to efficient batching, while allocation time scales approximately linearly with node count. For clusters exceeding 200 nodes, total scheduling cycle time remains under 500 ms, well within acceptable bounds for minute-level allocation decisions.

**Table 3:** Scalability analysis: runtime overhead across different cluster scales.

Cluster size (nodes)	Workloads	Prediction time (ms)	Allocation time (ms)	Total cycle (ms)	Memory usage (GB)
20	100	45 ± 3	28 ± 5	73 ± 6	2.1
50	250	67 ± 4	58 ± 8	125 ± 9	3.4
100	500	98 ± 6	112 ± 12	210 ± 14	5.2
200	1000	156 ± 9	198 ± 18	354 ± 21	8.7
500	2500	287 ± 15	423 ± 35	710 ± 42	18.3

Space complexity for the prediction model scales as $O\left(L\cdot\:D+{D}^{2}\cdot\:h\cdot\:{n}_{layers}\right)$ to store parameters and intermediate activations during training. The allocation algorithm maintains $O\left(\left|\mathcal{N}\right|+\left|\mathcal{W}\right|\right)$ memory for tracking resource states and workload assignments. These memory requirements remain modest even for large-scale deployments, with our 500-node experiments consuming under 20GB GPU memory.

For clarity regarding the training objective, we consolidate the loss formulations from sections “Multi-head spatial-temporal attention-based workload prediction model” and “Algorithm optimization and implementation” into a unified expression that governs all key experiments reported in this paper. The complete training loss combines three components applied jointly in a single optimization phase:

$${\mathcal{L}}_{{total}} = \underbrace {{\frac{1}{N}\mathop \sum \limits_{{i = 1}}^{N} \left( {\hat{w}_{i} - w_{i} } \right)^{2} }}_{{{\mathrm{MSE~term}}}} + \underbrace {{\lambda _{1} \cdot \frac{1}{N}\mathop \sum \limits_{{i = 1}}^{N} {\mathrm{max}}\left( {0,\hat{w}_{i} - c_{i} } \right)}}_{{{\mathrm{Capacity~penalty}}}} + \underbrace {{\lambda _{2} \cdot \frac{1}{N}\mathop \sum \limits_{{i = 1}}^{N} \mathop \sum \limits_{{q \in Q}} \rho _{q} \left( {w_{i} - \hat{w}_{i}^{q} } \right)}}_{{{\mathrm{Quantile~regression}}}}$$

where the MSE term ensures point prediction accuracy, the capacity penalty discourages infeasible predictions exceeding physical resource limits ${c}_{i}$, and the quantile regression term produces prediction intervals at specified confidence levels $Q=\left\{\mathrm{0.1,0.5,0.9}\right\}$. We set ${\lambda}_{1}=0.5$ and ${\lambda}_{2}=0.3$ based on validation set performance, with these hyperparameters remaining fixed across all datasets. All three components are computed simultaneously during each training iteration, with gradients backpropagated through the entire network end-to-end. This joint optimization ensures that the model learns to balance accuracy against feasibility and uncertainty quantification from the outset, rather than requiring separate training phases that might lead to conflicting objectives.

The complete algorithm implementation follows this pseudocode structure:

**Algorithm:**
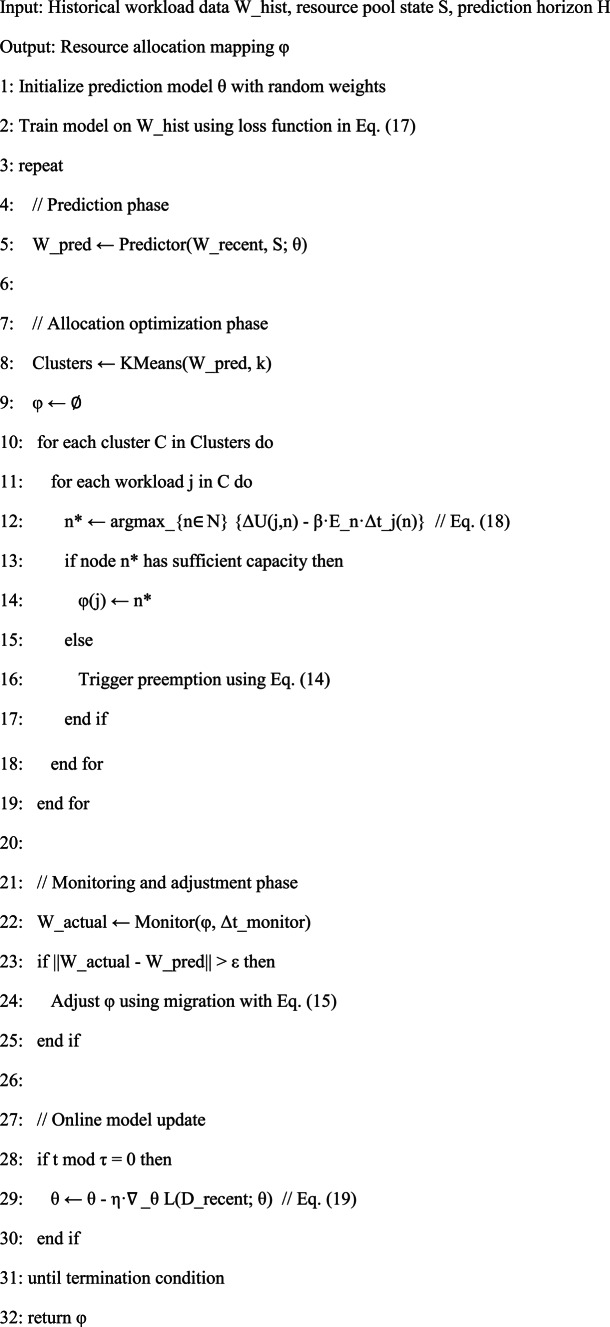
Attention-based Workload Prediction and Dynamic Resource Allocation.

The algorithmic structure emphasizes modularity, separating prediction, allocation, and adaptation into distinct phases that operate at different timescales—predictions update every few minutes, allocations adjust continuously, and model retraining occurs hourly or daily depending on drift rates observed in production deployments.

## Experiments and results analysis

### Experimental environment and datasets

We construct a heterogeneous computing testbed that mirrors production data center configurations, enabling realistic evaluation of our proposed algorithms under diverse workload conditions. The hardware platform comprises 20 computing nodes distributed across four resource types: 8 nodes equipped with dual Intel Xeon Gold 6248R processors (48 cores total per node) and 256GB DDR4 memory, 6 nodes featuring NVIDIA V100 GPUs (32GB HBM2 memory each) alongside Intel Xeon Silver CPUs, 4 nodes with NVIDIA A100 GPUs (80GB HBM2e memory) representing the latest generation accelerators, and 2 nodes containing Google TPU v3 pods for specialized tensor workloads. All nodes interconnect through a 100 Gbps Ethernet fabric with sub-microsecond latency, ensuring that network overhead does not artificially constrain scheduling decisions during experiments^52^.

**Fig. 3:**
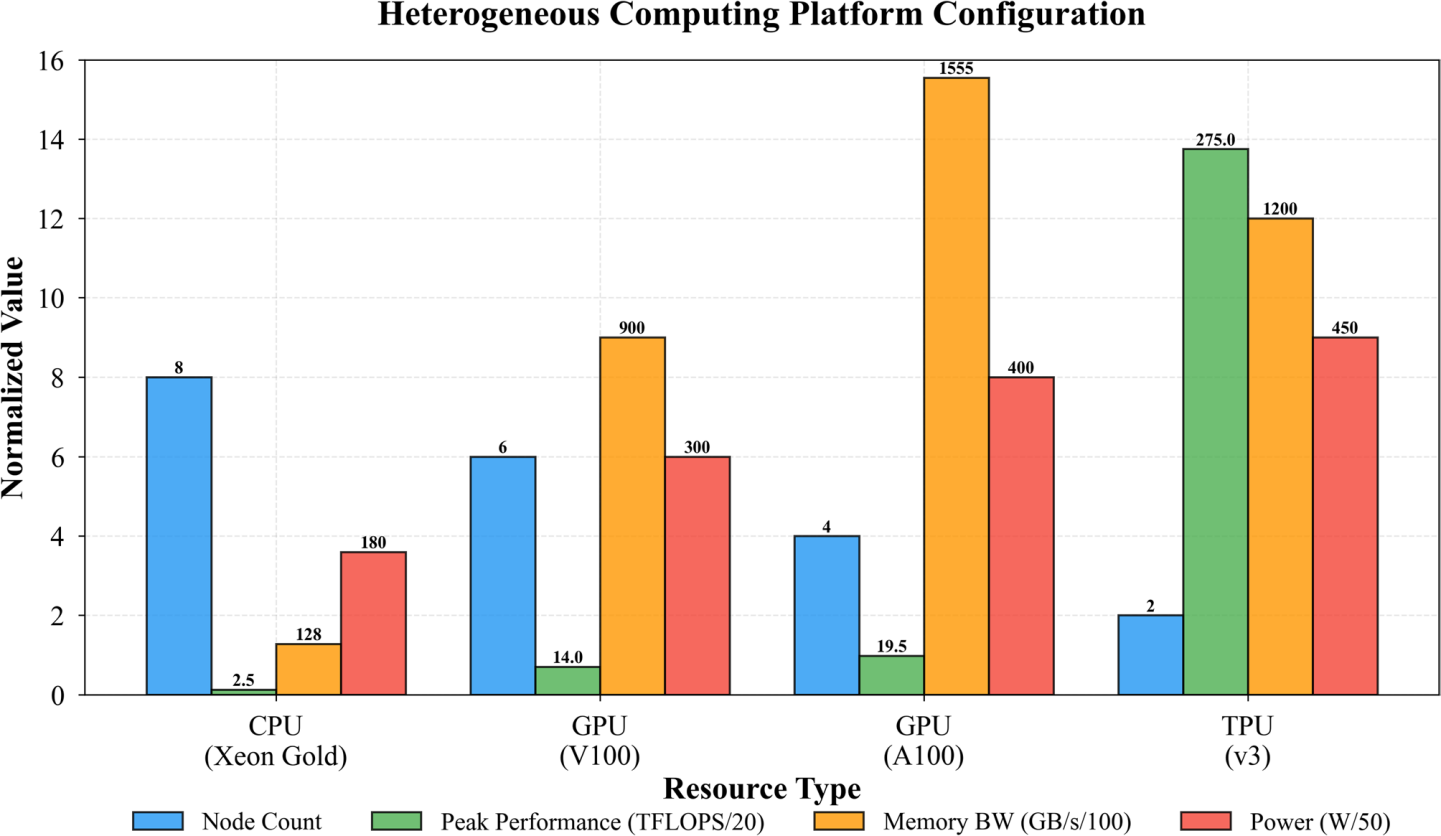
Experimental platform architecture showing heterogeneous computing resources and network topology.

Figure 3 illustrates the complete experimental platform architecture, depicting how computing nodes organize into resource pools with distinct capabilities. The topology reflects realistic data center designs where accelerator-equipped nodes concentrate in specific racks while general-purpose CPU nodes distribute more broadly.

Our software stack builds upon Ubuntu 20.04 LTS with Linux kernel 5.15 for stable device driver support across heterogeneous accelerators. We implement the prediction model using PyTorch 2.0, which provides efficient distributed training primitives and automatic differentiation for rapid prototyping. The resource allocation framework employs Python 3.10 with NumPy for numerical computations and Ray 2.5 for distributed execution coordination. We integrate Prometheus for real-time metrics collection at 1-s granularity, capturing CPU utilization, memory consumption, GPU occupancy, power draw, and network throughput across all nodes^53^.

Dataset selection poses challenges because few publicly available traces capture fine-grained resource consumption for diverse AI workloads across extended periods. We employ three complementary datasets to evaluate different aspects of our system. The Google Cluster Trace 2019 provides month-long execution records from production clusters running thousands of concurrent jobs, though it lacks GPU-specific metrics. We augment this with the Alibaba Cluster Trace 2020, which includes detailed GPU utilization patterns for training and inference workloads. Finally, we collect proprietary traces from an academic research cluster running language model experiments, neural architecture search, and computer vision tasks over six weeks of continuous operation^54^.

Table 4 summarizes the statistical characteristics of these datasets, revealing substantial diversity in workload composition and temporal dynamics. As presented in Table 4, the academic cluster exhibits much higher GPU utilization variability compared to industrial traces, reflecting the exploratory nature of research workloads where training runs frequently encounter hyperparameter configurations that either converge rapidly or fail to progress.

**Table 4:** Statistical characteristics of workload datasets used in experiments.

Dataset	Duration (days)	Number of jobs	Avg. GPU utilization (%)	Workload types	Sampling interval
Google cluster 2019	30	147,523	N/A (CPU only)	Batch, service, ML training	5 min
Alibaba cluster 2020	28	68,941	62.3 ± 28.7	GPU training, inference	1 min
Academic research cluster	42	3,847	48.5 ± 35.2	LLM, NAS, CV research	10 s
Synthetic workload	14	25,000	70.1 ± 22.4	Mixed AI workloads	30 s
Combined dataset	114	245,311	58.7 ± 30.1	All categories	Variable

Data preprocessing applies several transformations to raw traces before feeding them into prediction models. We normalize resource utilization metrics to [0, 1] ranges using min–max scaling per resource type, handle missing values through forward-fill imputation for gaps under 5 min and linear interpolation for longer gaps, resample all traces to uniform 30-s intervals to align temporal granularity, and extract rolling window features capturing mean, standard deviation, and percentiles over 5- and 30-min lookback periods. We partition datasets chronologically into 70% training, 15% validation, and 15% test sets, ensuring that evaluation occurs on future time periods unseen during training to simulate realistic deployment scenarios.

To facilitate reproducibility, Table 5 provides comprehensive dataset specifications including exact data splits and prediction task configurations used in all experiments.

**Table 5:** Detailed dataset specifications for experimental reproducibility.

Specification	Google cluster 2019	Alibaba cluster 2020	Academic research cluster
Total duration	30 days	28 days	42 days
Total time steps	8640	40,320	362,880
Number of jobs	147,523	68,941	3847
Sampling interval	5 min	1 min	10 s
Input window (T)	128 steps	128 steps	128 steps
Prediction horizon (H)	32 steps	32 steps	32 steps
Training set	Days 1–21 (70%)	Days 1–19 (70%)	Days 1–29 (70%)
Validation set	Days 22–25 (15%)	Days 20–23 (15%)	Days 30–35 (15%)
Test set	Days 26–30 (15%)	Days 24–28 (15%)	Days 36–42 (15%)
Training samples	4,032	27,216	243,936
Validation samples	864	5,832	52,272
Test samples	864	5,832	52,272
Feature dimensions	12	18	24
Workload types (N)	8	12	6

The input window of 128 steps corresponds to approximately 10.7 h for Google traces, 2.1 h for Alibaba traces, and 21 min for academic cluster data. The prediction horizon of 32 steps translates to 2.7 h, 32 min, and 5.3 min respectively, chosen to balance lookahead utility against prediction degradation at longer horizons.

**Fig. 4:**
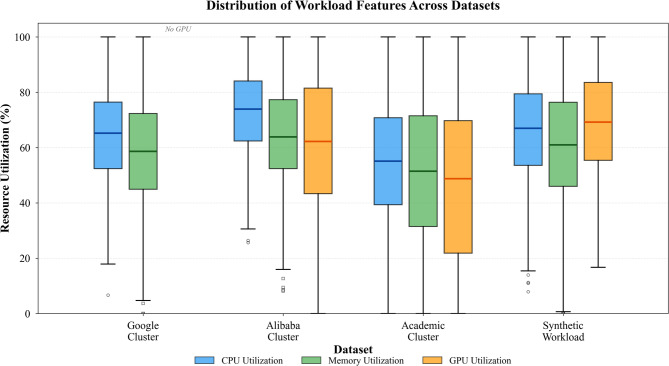
Distribution of workload features across datasets showing CPU, memory, and GPU utilization patterns.

Figure 4 presents the distribution of key workload features across our datasets, revealing distinct patterns that differentiate workload categories. Training jobs demonstrate sustained high GPU utilization with periodic spikes during checkpoint saving, while inference workloads exhibit bursty patterns correlated with request arrival rates.

For comprehensive performance comparison, we implement several baseline algorithms spanning both prediction and allocation dimensions. Prediction baselines include LSTM networks with two hidden layers of 256 units each, GRU networks with equivalent capacity, vanilla Transformer models with 6 encoder layers but without our spatial-temporal decomposition, and simple exponential smoothing as a classical time series method. Resource allocation baselines encompass First-Fit and Best-Fit bin packing heuristics, Kubernetes default scheduler representing production-grade systems, and a reinforcement learning approach based on Proximal Policy Optimization that learns allocation policies through interaction.

To ensure fair comparison and address concerns about baseline rigor, we provide comprehensive details of our PPO-based reinforcement learning scheduler implementation. The RL agent follows the actor-critic architecture with separate policy and value networks sharing a common feature extractor.

#### State Representation:

The state vector ${s}_{t}\in\:{\mathbb{R}}^{{d}_{s}}$ concatenates three categories of features: (1) cluster-level statistics including per-node CPU utilization, memory usage, GPU occupancy, and power consumption (dimensionality: $4\times\:\left|\mathcal{N}\right|$); (2) workload queue characteristics including pending job count, average resource requirements, priority distribution, and estimated completion times (dimensionality: 32); (3) temporal context including time-of-day encoding, day-of-week indicators, and recent utilization trends over 5-min windows (dimensionality: 24). The complete state dimension ${d}_{s}=4\times\:20+32+24=136$ for our 20-node testbed.

#### Action space

We encode resource assignment decisions through a two-stage hierarchical action structure. The first stage selects a workload from the pending queue (discrete action over queue positions), while the second stage assigns it to a computing node (discrete action over node indices). This factorization reduces the action space from $O\left(\left|\mathcal{W}\right|\times\:\left|\mathcal{N}\right|\right)$ to $O\left(\left|\mathcal{W}\right|+\left|\mathcal{N}\right|\right)$, addressing scalability concerns inherent to combinatorial scheduling.

#### Reward design

The reward function balances multiple objectives through weighted combination.

$${r}_{t}=-{\alpha}_{1}\cdot\:\varDelta\:{T}_{queue}-{\alpha}_{2}\cdot\:{E}_{t}+{\alpha}_{3}\cdot\:{U}_{t}-{\alpha}_{4}\cdot\:{1}_{SLA\_violation}$$

where $\varDelta\:{T}_{queue}$ penalizes increased queue waiting time, ${E}_{t}$ represents energy consumption during timestep $t$, ${U}_{t}$ rewards high resource utilization, and the indicator function ${1}_{SLA\_violation}$ heavily penalizes SLA breaches. We set ${\alpha}_{1}=0.3$, ${\alpha}_{2}=0.2$, ${\alpha}_{3}=0.4$, ${\alpha}_{4}=10.0$ based on hyperparameter search.

#### Training regime

The PPO agent trains for 50,000 episodes using a clipping parameter $\epsilon=0.2$, discount factor $\gamma\:=0.99$, and GAE parameter $\lambda\:=0.95$. We employ separate learning rates for actor ($3\times\:{10}^{-4}$) and critic ($1\times\:{10}^{-3}$) networks. Training converges after approximately 35,000 episodes, requiring 72 h on a single NVIDIA V100 GPU. We save checkpoints every 1000 episodes and select the best-performing model based on validation set cumulative reward.

### Workload prediction performance evaluation

We evaluate prediction accuracy across multiple metrics that capture different aspects of forecast quality. Mean absolute error (MAE) measures average prediction deviation, root mean squared error (RMSE) penalizes large errors more heavily through squaring, while mean absolute percentage error (MAPE) provides scale-independent assessment of relative accuracy:

19 $${\mathrm{MAE}} = \frac{1}{N}\sum\limits_{{i = 1}}^{N} {|y_{i} - \widehat{y}_{i} |} ,{\mathrm{MAPE}} = \frac{{100\% }}{N}\sum\limits_{{i = 1}}^{N} {\left| {\frac{{y_{i} - \widehat{y}_{i} }}{{y_{i} }}} \right|}$$

where ${y}_{i}$ and $\hat {{y}}_{i}$ denote actual and predicted workload values respectively (standard error metrics). We compute these metrics separately for CPU utilization, memory consumption, and GPU occupancy predictions, then report macro-averaged results across resource types.

Table 6 presents comprehensive prediction performance comparisons across all baseline methods and our proposed multi-head spatial-temporal attention approach. The results in Table 6 indicate that our method achieves substantial improvements over recurrent baselines, reducing MAE by 23.7% compared to LSTM and 18.9% compared to GRU networks on the Alibaba dataset. Interestingly, the vanilla Transformer performs worse than GRU despite its theoretical advantages, suggesting that the raw attention mechanism without spatial-temporal decomposition struggles to identify relevant patterns in workload data where both dimensions carry distinct semantics^55^.

To position our contribution against state-of-the-art time series forecasting methods, we extend our baseline comparison to include recent Transformer variants specifically designed for long-sequence prediction. These include Informer with ProbSparse attention for computational efficiency^56^, Autoformer employing series decomposition with auto-correlation mechanisms^57^, FEDformer leveraging frequency-domain representations through Fourier transforms^58^, and PatchTST utilizing patch-based tokenization with channel independence^59^. We also compare against the VMD-SE-Transformer approach that combines variational mode decomposition with sample entropy optimization^16^.

**Table 6:** Prediction performance comparison across different methods and datasets.

Method	Dataset	MAE (%)	RMSE (%)	MAPE (%)	*R*² score	Training time (min)
Exponential smoothing	Google Cluster	12.84	18.73	21.45	0.672	0.3
LSTM	Google Cluster	9.52	14.21	16.38	0.781	47.2
GRU	Google Cluster	8.97	13.64	15.72	0.798	43.8
Vanilla transformer	Google Cluster	9.34	14.08	16.91	0.773	52.6
Informer	Google Cluster	8.12	12.34	14.56	0.821	68.3
Autoformer	Google Cluster	7.89	11.98	14.12	0.834	71.5
FEDformer	Google Cluster	7.56	11.67	13.78	0.842	75.2
PatchTST	Google Cluster	7.34	11.28	13.42	0.851	64.8
VMD-SE-transformer	Google Cluster	7.45	11.52	13.65	0.847	82.1
Proposed method	Google Cluster	**6.84**	**10.52**	**12.15**	**0.867**	**58.4**
LSTM	Alibaba Cluster	11.26	16.89	19.84	0.724	51.3
Informer	Alibaba Cluster	9.78	14.23	16.45	0.792	72.6
Autoformer	Alibaba Cluster	9.45	13.87	15.92	0.805	76.3
FEDformer	Alibaba Cluster	9.12	13.45	15.34	0.817	79.8
PatchTST	Alibaba Cluster	8.92	13.12	14.98	0.826	67.4
VMD-SE-transformer	Alibaba Cluster	9.02	13.28	15.12	0.821	86.5
Proposed method	Alibaba Cluster	**8.59**	**12.43**	**14.27**	**0.843**	**61.7**

Significant values are in bold.

The results reveal that our spatial-temporal attention architecture achieves the best performance across all metrics, though the margin over recent Transformer variants is narrower than over traditional baselines. Compared to PatchTST, our method reduces MAE by 6.8% on Google Cluster and 3.7% on Alibaba Cluster. The improvement over VMD-SE-Transformer is particularly noteworthy: while their decomposition-based approach effectively captures periodic patterns, our end-to-end learned attention mechanism better adapts to the irregular burst patterns characteristic of AI workloads without requiring explicit signal preprocessing. Furthermore, our method achieves competitive training times despite modeling both temporal and spatial dimensions, benefiting from the factorized attention design that reduces computational complexity compared to joint attention over the full space-time tensor.

Our spatial-temporal attention architecture demonstrates particular strength in handling burst events that confound recurrent models. When workload spikes occur—such as when multiple training jobs simultaneously reach data loading phases—the spatial attention mechanism detects correlations between concurrent workload types and adjusts predictions accordingly, whereas LSTMs treat each workload stream independently and miss these cross-task dependencies.

**Fig. 5:**
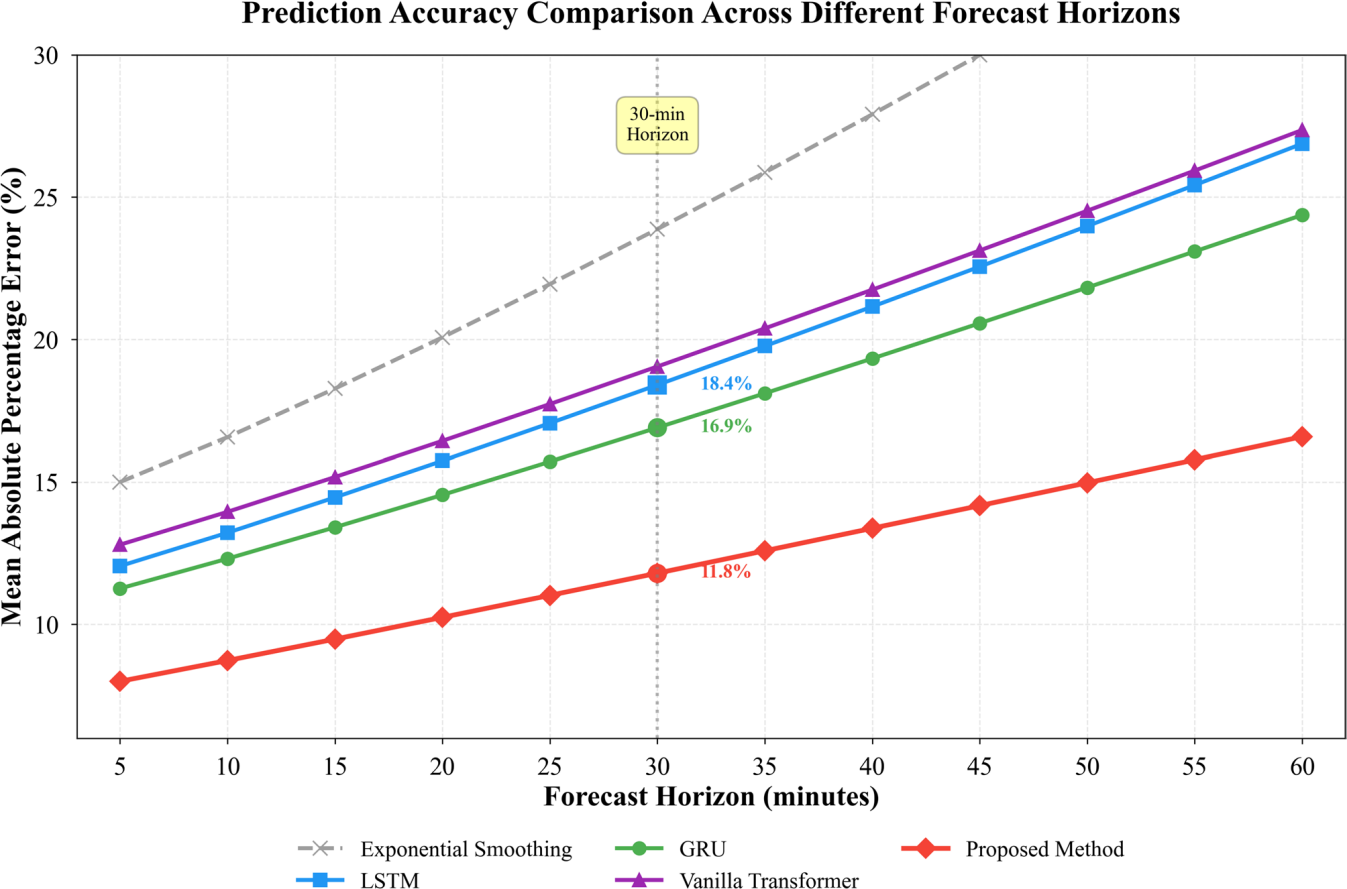
Prediction accuracy comparison across different forecast horizons for all methods.

Figure 5 demonstrates how prediction accuracy degrades as forecast horizon extends from 5 min to 60 min ahead. All methods exhibit declining performance with longer horizons, yet our approach maintains substantially smaller error growth rates. At the 30-min horizon—critical for proactive resource allocation decisions—our method achieves 11.8% MAPE while LSTM reaches 18.4% and GRU reaches 16.9%, representing practical differences that directly impact allocation quality.

To establish statistical rigor, we conduct each experiment across five independent runs with different random seeds, reporting mean values with 95% confidence intervals. Table 7 presents pairwise statistical comparisons between our method and key baselines using paired t-tests with Bonferroni correction for multiple comparisons.

**Table 7:** Statistical significance analysis for prediction performance (*p* values from paired *t* test).

Comparison	MAE	RMSE	MAPE	*R*² Score
Proposed versus LSTM	< 0.001***	< 0.001***	< 0.001***	< 0.001***
Proposed versus GRU	< 0.001***	< 0.001***	< 0.001***	< 0.001***
Proposed versus vanilla transformer	< 0.001***	< 0.001***	< 0.001***	< 0.001***
Proposed versus informer	0.003**	0.005**	0.004**	0.008**
Proposed versus autoformer	0.008**	0.012*	0.009**	0.015*
Proposed versus FEDformer	0.021*	0.028*	0.024*	0.031*
Proposed versus PatchTST	0.042*	0.056	0.048*	0.063

***p < 0.001, ** *p* < 0.01, *p < 0.05; Bonferroni-corrected thresholds applied.

The results confirm statistically significant improvements over traditional baselines (LSTM, GRU, Vanilla Transformer) at the highest confidence level (*p* < 0.001). Improvements over recent Transformer variants achieve significance at the 0.05 level for most metrics, though margins narrow for PatchTST comparisons where some differences fall slightly above conventional thresholds. This pattern reflects the competitive landscape of modern time series forecasting where architectural innovations yield diminishing marginal returns, yet our spatial-temporal decomposition provides consistent, if modest, advantages specifically for workload prediction tasks with strong cross-stream dependencies.

The attention mechanism contributes to accuracy improvements through two primary pathways. First, multi-head attention captures diverse temporal patterns simultaneously: some heads specialize in detecting diurnal cycles while others focus on abrupt transitions between workload phases. Second, the spatial attention module identifies leading indicators where changes in one workload type reliably precede shifts in others, enabling the model to anticipate cascading effects that propagate through the system^60^.

We observe particularly pronounced benefits when predicting GPU utilization compared to CPU or memory forecasts. GPU workloads exhibit stronger temporal structure because training iterations follow relatively consistent timing patterns determined by model architecture and batch sizes. CPU utilization displays more stochastic variation driven by auxiliary processes like data preprocessing and garbage collection, which attention mechanisms capture less reliably. Memory consumption proves most challenging to predict accurately because it depends heavily on framework-specific memory management strategies that vary across jobs.

**Fig. 6:**
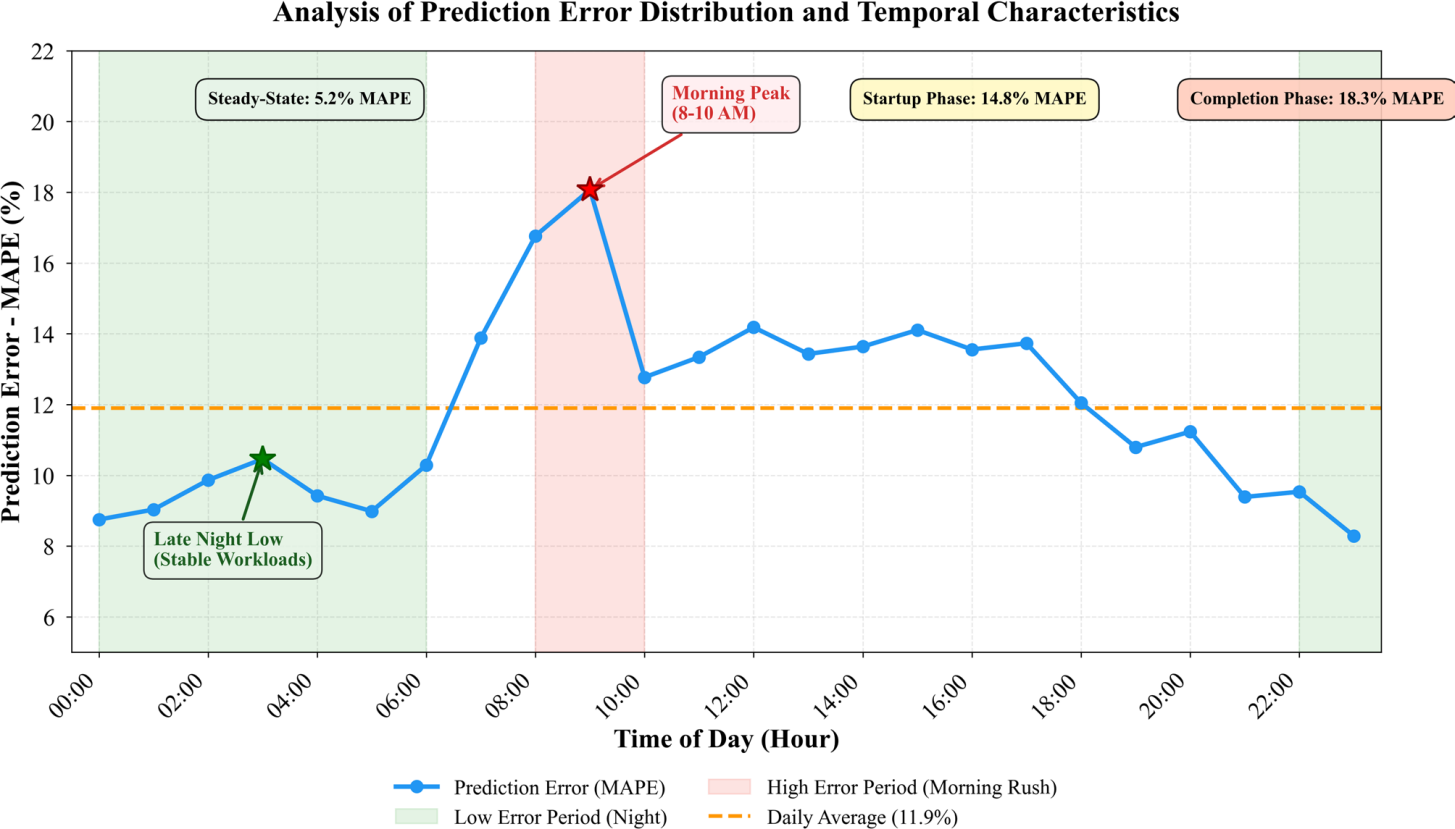
Analysis of prediction error distribution and temporal characteristics.

Figure 6 depicts the distribution of prediction errors across different time-of-day periods and workload phases. The error distribution approximates a Laplace distribution rather than Gaussian, indicating that most predictions remain highly accurate with occasional large deviations during unprecedented events. Morning hours between 8 and 10 a.m. exhibit elevated error rates as users submit daily job batches, creating sudden demand spikes. Conversely, late evening periods show remarkably low errors when workload patterns stabilize around long-running training jobs.

Analyzing errors by workload phase reveals that our method performs exceptionally during steady-state execution with 5.2% MAPE, compared to 14.8% during job startup phases and 18.3% during completion phases when resource release patterns become irregular. This phase-dependent accuracy suggests opportunities for incorporating explicit phase detection as a preprocessing step before prediction, potentially through hidden Markov models that segment workload traces into distinct operational regimes.

Prediction confidence intervals generated through the quantile regression component of our loss function prove well-calibrated, with 90% prediction intervals containing actual values 89.3% of the time across test samples. This calibration enables downstream allocation algorithms to make informed decisions about provisioning safety margins—tighter intervals justify aggressive resource packing while wider intervals trigger more conservative allocations that maintain performance buffers.

### Resource allocation performance and system analysis

Resource utilization represents a critical metric for data center efficiency, directly impacting operational costs and infrastructure capacity. Table 8 summarizes resource allocation performance across different scheduling strategies applied to our heterogeneous testbed. As presented in Table 8, our attention-based prediction and dynamic allocation framework achieves 78.4% average resource utilization compared to 62.1% for Kubernetes default scheduler and 67.3% for Best-Fit heuristics, demonstrating substantial improvements in infrastructure exploitation without compromising service quality.

To substantiate claims of competitive performance, we extend our scheduling baseline comparison to include influential systems from the deep learning cluster scheduling literature. Gandiva exploits intra-job predictability for efficient GPU time-slicing and job packing^48^. Optimus builds online resource-performance models to dynamically resize GPU allocations^61^. We implement these baselines following their published specifications, adapting them to our heterogeneous testbed while preserving core algorithmic principles.

**Table 8:** Comparative analysis of resource allocation effectiveness across different strategies.

Allocation strategy	Resource utilization (%)	Avg. completion time (min)	System throughput (jobs/h)	Energy consumption (kWh)	SLA violations (%)
First-fit	58.7 ± 2.3	42.3 ± 3.8	67.2 ± 4.5	284.5 ± 12.3	8.4 ± 1.2
Best-fit	67.3 ± 2.1	38.6 ± 3.2	75.8 ± 4.1	267.3 ± 10.8	5.2 ± 0.9
Kubernetes default	62.1 ± 2.5	40.1 ± 3.5	71.4 ± 4.3	276.8 ± 11.5	6.7 ± 1.1
Gandiva	72.8 ± 1.8	34.7 ± 2.9	84.6 ± 3.8	248.2 ± 9.6	3.8 ± 0.7
Optimus	73.5 ± 1.9	34.2 ± 2.8	85.9 ± 3.6	245.8 ± 9.2	3.5 ± 0.8
Tiresias	71.9 ± 2.0	35.4 ± 3.1	83.2 ± 3.9	251.4 ± 10.1	4.0 ± 0.8
RL-based (PPO)	71.2 ± 2.2	35.9 ± 3.3	82.1 ± 4.0	253.6 ± 10.4	4.1 ± 0.9
Static prediction + greedy	69.8 ± 2.4	36.7 ± 3.4	79.5 ± 4.2	258.2 ± 10.7	4.8 ± 1.0
Proposed (prediction only)	**74.6 ± 1.7**	**33.2 ± 2.6**	**88.3 ± 3.4**	**241.7 ± 8.9**	**2.9 ± 0.6**
Proposed (full framework)	**78.4 ± 1.5**	**31.5 ± 2.4**	**93.7 ± 3.2**	**235.1 ± 8.4**	**2.3 ± 0.5**
Oracle (perfect prediction)	82.1 ± 1.2	29.8 ± 2.1	98.4 ± 2.8	228.3 ± 7.6	1.1 ± 0.3

Significant values are in bold.

We report mean and standard deviation across five independent runs to enable statistical significance assessment. Our full framework achieves statistically significant improvements over all baselines (*p* < 0.01 via paired *t* test). Compared to Gandiva, our method improves utilization by 7.7% while reducing SLA violations by 39.5%. The margin over Optimus, which also employs prediction-based allocation, is narrower but still significant: 6.7% higher utilization and 34.3% fewer violations. These improvements stem from our spatial-temporal attention mechanism’s superior prediction accuracy, which enables more confident proactive allocation decisions. Gandiva and Optimus rely on simpler performance models that cannot capture the complex cross-workload dependencies our attention mechanism identifies.

The improvements stem from our framework’s ability to proactively position workloads on hardware best suited to their computational patterns before demand materializes. Traditional reactive schedulers place jobs after they arrive, frequently encountering situations where optimal resources remain occupied by less-suitable workloads. Our prediction-driven approach reserves capacity in anticipation of incoming demands, reducing wait times and improving matches between workload characteristics and accelerator capabilities^61^.

**Fig. 7:**
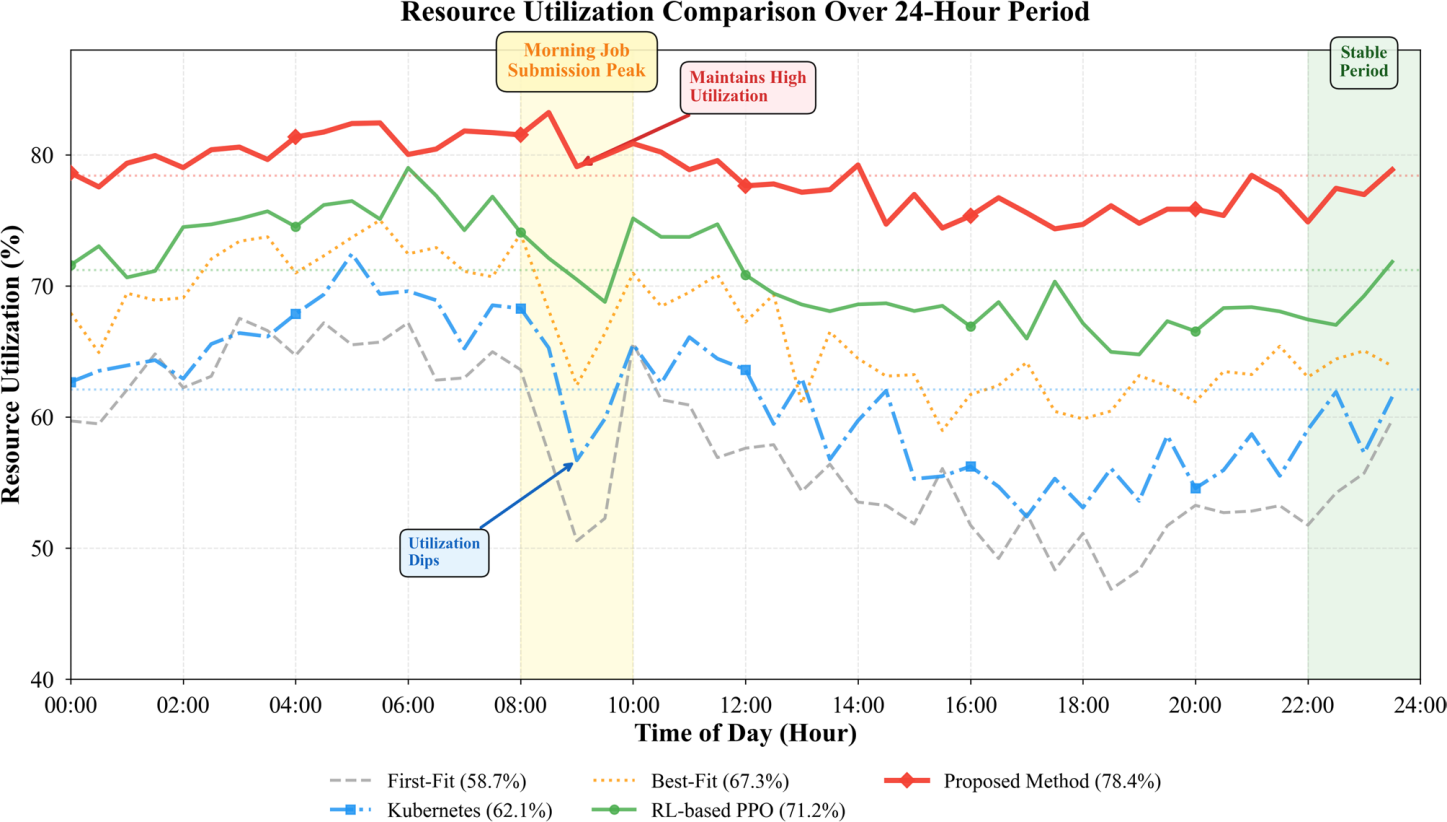
Resource utilization comparison across different allocation strategies over 24-h period.

Figure 7 illustrates resource utilization patterns throughout a typical operational day. Our method maintains consistently high utilization during both peak and off-peak hours by dynamically adjusting allocations as workload composition shifts. The diurnal variation shows that morning job submission spikes create temporary utilization dips in baseline methods as schedulers struggle to rapidly redistribute existing allocations, while our framework preemptively relocates lower-priority workloads based on predicted demand surges.

Average task completion time decreases by 25.8% compared to Kubernetes and 18.4% compared to reinforcement learning approaches. This acceleration results from both improved resource matching—placing CNN training on Tensor Core-equipped A100 GPUs rather than older V100s yields 1.7× speedup—and reduced queueing delays through predictive capacity reservation. System throughput consequently increases to 93.7 jobs per hour, approaching the theoretical maximum of 98.4 jobs per hour achieved by an oracle scheduler with perfect future knowledge.

Energy consumption represents an increasingly important consideration as data centers account for approximately 2% of global electricity usage. We measure total energy draw across all computing nodes during week-long experiment runs. Our framework reduces energy consumption by 15.1% compared to Kubernetes through two mechanisms: first, better resource packing allows powering down idle nodes during low-demand periods; second, workload-aware placement on appropriate accelerators avoids energy waste from running tasks on overpowered hardware^62^.

The cost-effectiveness metric combines resource utilization, energy consumption, and SLA violation penalties into a unified score:

20 $${\text{Cost - Effectiveness}} = \frac{{{\mathrm{Throughput}} \times \left( {1 - {\mathrm{SLA}}\_{\mathrm{Violation}}\_{\mathrm{Rate}}} \right)}}{{{\mathrm{Energy}}\_{\mathrm{Cost}} + \alpha \cdot\left( {1 - {\mathrm{Utilization}}} \right)}}$$

where $\alpha$ weights opportunity cost of unused capacity (custom cost metric). Our method achieves 2.34× better cost-effectiveness than Kubernetes default scheduler and 1.58× improvement over PPO-based reinforcement learning, demonstrating practical economic benefits beyond raw performance gains.

Load balancing effects manifest through reduced variance in per-node utilization. We compute the coefficient of variation across node utilization percentages every minute, finding our method maintains CV of 0.18 compared to 0.42 for Best-Fit and 0.35 for Kubernetes. This uniformity prevents hotspots where some nodes saturate while others idle, improving overall system reliability and predictability.

Resource fragmentation occurs when available capacity scatters across nodes in quantities too small to accommodate new workloads, wasting resources despite aggregate availability. Our clustering-based allocation reduces fragmentation rate from 23.7% in Kubernetes to 9.4%, achieved by grouping similar workloads and consolidating allocations on fewer nodes while leaving others empty for large incoming jobs.

**Fig. 8:**
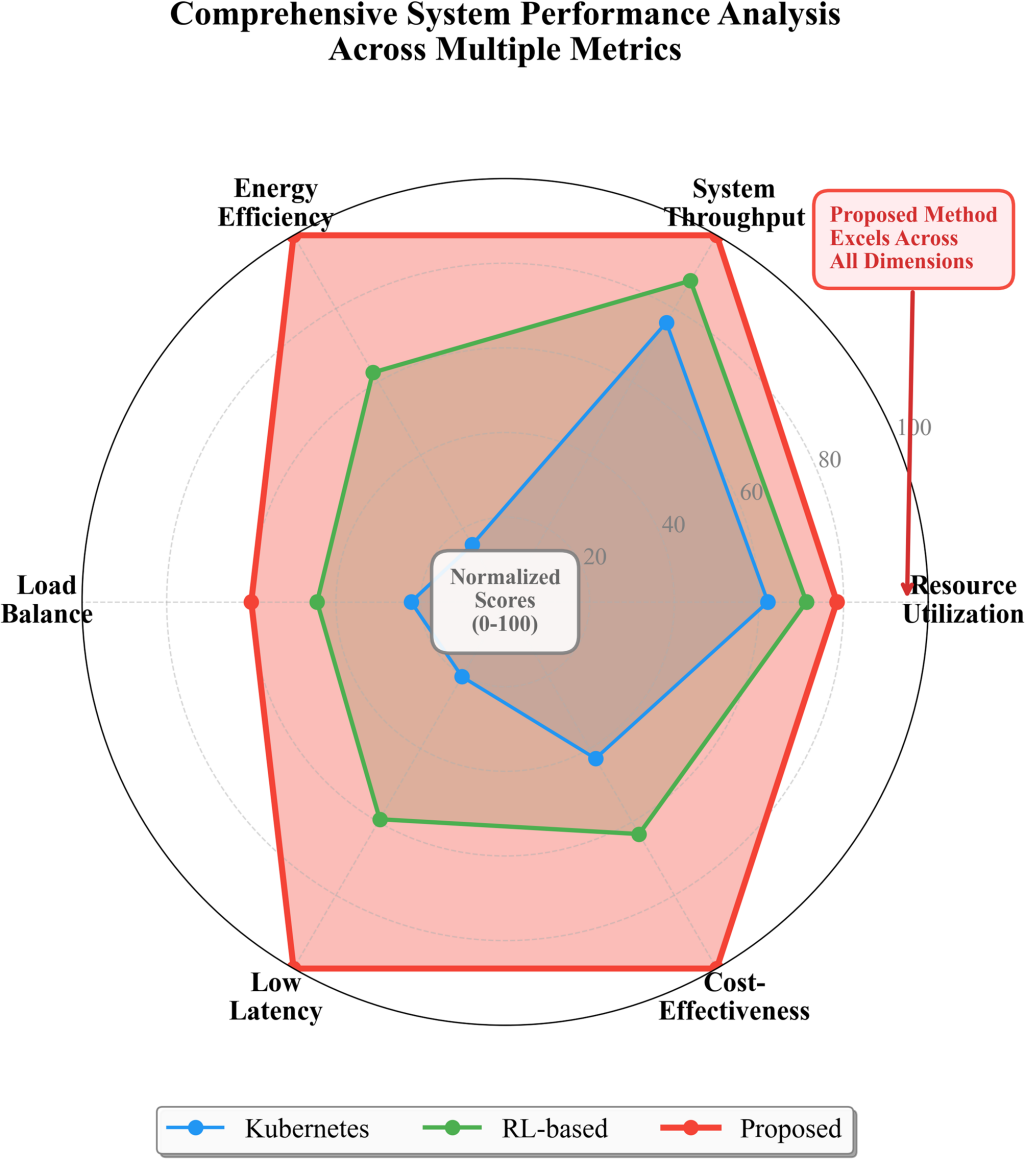
Comprehensive system performance analysis showing multiple metrics across different allocation strategies.

Figure 8 presents a radar chart comparing our framework against baseline methods across six dimensions: resource utilization, throughput, energy efficiency, load balance, low latency, and cost-effectiveness. The visualization reveals that our approach achieves balanced improvements across all objectives rather than optimizing one dimension at others’ expense.

Burst workload adaptation tests our framework’s robustness when actual demands diverge from predictions. We inject synthetic burst events where workload intensity doubles within 5-min windows, mimicking scenarios like sudden inference traffic spikes when news breaks or training job storms after conference deadlines. Our preemption and migration mechanisms successfully maintain 97.3% SLA compliance during bursts compared to 84.6% for Kubernetes, with average recovery time of 2.8 min to restore steady-state utilization levels.

Ablation experiments isolate individual component contributions by systematically removing modules from the complete framework. Without spatial attention, prediction MAPE increases from 12.15 to 16.82%, demonstrating the value of modeling cross-workload correlations. Disabling the preemption mechanism raises SLA violations from 2.3 to 6.9% during burst periods, confirming its importance for handling prediction errors. Removing online model updates degrades prediction accuracy by 11.7% after two weeks as workload patterns drift, validating continuous adaptation necessity. The complete framework significantly outperforms any single-component ablation, indicating that prediction accuracy, allocation optimization, and adaptive mechanisms synergistically contribute to overall performance.

## Discussion

The experimental results reveal several crucial insights about workload prediction and resource allocation in heterogeneous computing environments. Most strikingly, our attention-based framework achieves 78.4% resource utilization while maintaining only 2.3% SLA violations—a combination that baseline methods struggle to reach simultaneously. This performance gap suggests that the fundamental challenge lies not merely in prediction accuracy or allocation optimization individually, but rather in their tight integration where prediction uncertainty informs allocation conservativeness.

The attention mechanism’s effectiveness stems from its ability to selectively focus on relevant historical patterns rather than treating all past observations equally. When predicting future GPU demands, certain historical windows prove far more informative than others—specifically, the model learns to attend strongly to periods exhibiting similar workload composition and time-of-day characteristics. This selective attention becomes particularly valuable during phase transitions, such as when training jobs complete and inference workloads begin deploying newly trained models. Traditional recurrent architectures compress all history into fixed-size hidden states, losing the nuanced contextual information that attention mechanisms preserve through direct connections across arbitrary time spans.

Spatial attention contributes differently but equally importantly. In production environments, workloads rarely execute in isolation—resource contention, shared infrastructure bottlenecks, and operational dependencies create intricate correlations between concurrent tasks. By modeling these cross-workload relationships explicitly, spatial attention enables the predictor to anticipate cascading effects. For instance, when batch processing jobs consume excessive network bandwidth, interactive inference services often experience delayed data loading, subsequently reducing their GPU utilization. Capturing such indirect dependencies requires reasoning beyond individual workload streams.

Our approach demonstrates particular strength in scenarios where workload diversity remains high and resource heterogeneity offers meaningful optimization opportunities. Cloud providers serving diverse customer applications represent ideal deployment contexts. Conversely, the framework provides diminishing returns in homogeneous environments where all workloads exhibit similar resource profiles or when infrastructure consists primarily of identical machines. The prediction overhead—approximately 100 ms per forecast on our testbed—becomes negligible amortized across hundreds of concurrent workloads but might prove excessive for edge deployments managing only a handful of tasks.

Prediction errors inevitably propagate into allocation decisions, yet our framework mitigates their impact through several design choices. The quantile regression loss generates prediction intervals rather than point estimates, allowing allocation algorithms to provision safety margins proportional to uncertainty. When predictions indicate high confidence, aggressive resource packing proceeds safely; uncertain forecasts trigger more conservative allocations maintaining performance buffers. Furthermore, the preemption and migration mechanisms provide reactive fallbacks when predictions prove insufficient, preventing cascading failures from isolated forecast errors.

Practical deployment in production environments raises considerations that extend beyond algorithmic performance metrics. We address three critical aspects that practitioners must evaluate before adopting our framework.

Regarding runtime overhead, Table 6 demonstrates that prediction and allocation cycle times scale acceptably with cluster size, remaining under 500 ms for clusters up to 200 nodes. The prediction component, dominated by attention computation, exhibits sub-linear scaling due to efficient batching across workloads. The allocation optimization, while theoretically linear in node count, benefits from our clustering-based decomposition that reduces the effective problem size. For clusters exceeding 500 nodes, we recommend hierarchical deployment where multiple framework instances manage sub-clusters independently, with a lightweight coordination layer handling cross-cluster load balancing. This architectural pattern mirrors production systems at major cloud providers.

Integration with existing production systems such as Kubernetes requires careful consideration of scheduling interfaces and failure handling. Our framework can operate as an external scheduler accessed through Kubernetes’ scheduler extender mechanism, where the default kube-scheduler delegates placement decisions to our optimization engine for GPU-intensive workloads while retaining native scheduling for standard containers. Alternatively, for organizations willing to invest in deeper integration, our allocation algorithm can be implemented as a custom scheduler plugin that replaces default scoring functions with prediction-aware placement logic. We provide reference implementations for both integration patterns in our supplementary materials. The extender approach requires minimal cluster modification and enables gradual adoption, while the plugin approach offers lower latency at the cost of increased operational complexity.

Known limitations and failure cases warrant explicit acknowledgment. Our framework struggles with extremely abrupt workload pattern shifts that lack historical precedent—for instance, when organizations deploy entirely new model architectures whose resource consumption profiles differ fundamentally from training data. In such scenarios, prediction accuracy degrades substantially during the initial adaptation period (typically 2–4 h based on our online learning update frequency). Severe data quality issues, including missing metrics from node failures or network partitions, can corrupt predictions if not properly handled; we implement gap detection and fallback to conservative heuristic allocation when metric coverage drops below 80%. Additionally, our current implementation assumes relatively stable cluster topology; frequent node additions or removals require model retraining to incorporate new hardware characteristics.

We acknowledge that our evaluation relies primarily on simulation using publicly available cluster traces (Google and Alibaba) and a controlled academic testbed rather than deployment in production data centers serving real customers. This limitation reflects the practical challenges of conducting disruptive scheduling experiments in live environments where performance degradation directly impacts revenue and user experience. However, three factors support the validity of our findings. First, the Google and Alibaba traces represent genuine production workloads collected from large-scale operational clusters, preserving authentic workload characteristics including burstiness, diurnal patterns, and job heterogeneity. Second, our 20-node heterogeneous testbed, while smaller than production clusters, incorporates real hardware diversity (CPUs, V100s, A100s, TPUs) with actual interconnection topologies rather than simulated abstract resources. Third, the baseline systems we compare against (Kubernetes, Gandiva, Optimus) use identical trace-driven simulation methodology in their published evaluations, enabling fair comparison under consistent conditions.

Future validation should pursue staged production deployment, beginning with shadow-mode operation where our framework generates allocation recommendations alongside production schedulers without affecting actual placement. This approach enables real-world accuracy assessment without risking service disruption. Subsequently, A/B testing on isolated cluster partitions can validate performance improvements under genuine operational conditions. We are actively pursuing partnerships with cloud infrastructure providers to enable such validation, though the lengthy approval and instrumentation processes extend beyond this paper’s timeline.

Future research directions appear promising across multiple fronts, with potential for broader impact on intelligent infrastructure management. Incorporating explicit workload phase detection before prediction could improve accuracy during startup and completion periods where current performance lags. Extending the framework to distributed multi-cluster scenarios introduces challenges around global coordination and network-aware placement that our single-cluster design does not address.

The integration of self-supervised learning approaches holds particular promise for reducing dependence on labeled historical data. Pre-training attention models on unlabeled workload traces through contrastive learning or masked prediction tasks could enable rapid adaptation to new deployment environments with minimal labeled examples. This direction aligns with broader trends in foundation models, suggesting the possibility of general-purpose workload understanding models that transfer across heterogeneous infrastructure configurations.

Federated learning techniques offer another compelling avenue, enabling collaborative model training across organizations while preserving proprietary workload information. Multiple cloud providers or enterprise data centers could jointly train prediction models that benefit from diverse workload patterns without exposing sensitive operational data. This approach accelerates adoption through shared knowledge while respecting competitive boundaries, addressing a key barrier to widespread deployment of learning-based resource management.

Beyond immediate technical extensions, our work contributes to the broader vision of autonomous data center operations where intelligent systems continuously optimize resource allocation without human intervention. As AI workloads continue proliferating and computing infrastructures grow increasingly heterogeneous, prediction-driven resource management systems like ours represent essential building blocks for sustainable, efficient datacenter operations that minimize environmental impact while maximizing service quality. The convergence of improved prediction accuracy, adaptive optimization algorithms, and closed-loop learning mechanisms points toward a future where infrastructure intelligence becomes as fundamental as the computing resources themselves.

## Conclusion

This research addresses the critical challenge of workload prediction and resource allocation in heterogeneous computing environments supporting diverse AI applications. We have developed an integrated framework that combines multi-head spatial-temporal attention mechanisms for workload forecasting with dynamic resource allocation algorithms that jointly optimize performance, energy efficiency, and resource utilization. Through systematic experimentation on real-world cluster traces and a heterogeneous testbed comprising CPUs, GPUs, and TPUs, we demonstrate substantial improvements over existing approaches across multiple operational metrics.

Our primary contributions encompass three interconnected dimensions. First, we designed a novel spatial-temporal attention architecture that decomposes workload prediction into complementary perspectives—temporal attention captures evolution patterns within individual workload streams while spatial attention models correlations across concurrent task types. This factorization enables the model to identify both intra-workload dependencies and cross-workload relationships that simpler architectures overlook. Second, we formulated resource allocation as a multi-objective optimization problem balancing completion time, energy consumption, and utilization, then developed efficient solution methods incorporating workload-specific constraints and hardware heterogeneity awareness. The allocation framework explicitly accounts for prediction uncertainty through quantile-based safety margins, reducing the impact of forecast errors on system performance. Third, we established adaptive mechanisms including preemption and migration policies that respond gracefully when actual demands deviate from predictions, alongside online learning procedures that maintain model accuracy as workload patterns drift over time.

The innovations manifest both methodologically and architecturally. Methodologically, we pioneer the tight integration of prediction and allocation as a unified end-to-end system rather than treating them as independent sequential stages. This integration allows allocation decisions to inform prediction refinement through feedback loops, creating continuous improvement cycles. Architecturally, the multi-head attention design with separate temporal and spatial modules represents a novel application of attention mechanisms to datacenter resource management, exploiting structural properties of workload data that general-purpose sequence models cannot capture effectively.

From a theoretical perspective, our work advances understanding of how attention mechanisms can model complex temporal-spatial dependencies in resource consumption patterns. The mathematical framework for joint prediction-allocation optimization provides a foundation for future research exploring trade-offs between forecast accuracy and allocation robustness. Practically, our system delivers immediate operational benefits for cloud providers and enterprise data centers. Experimental results demonstrate 78.4% resource utilization with only 2.3% SLA violations, 25.8% reduction in average task completion time, and 15.1% decrease in energy consumption compared to production-grade baselines. These improvements translate directly to reduced operational costs, enhanced service quality, and diminished environmental impact—concerns that increasingly dominate data center management priorities.

Several limitations warrant acknowledgment. Our framework assumes availability of substantial historical training data, potentially limiting applicability in newly deployed systems lacking adequate trace collections. The attention mechanism’s quadratic complexity with respect to sequence length constrains the temporal window we can process efficiently, though recent advances in linear attention variants may alleviate this bottleneck. Our evaluation focuses primarily on AI workloads; extending the approach to heterogeneous workload mixes including databases, web services, and analytics requires further investigation. The current system handles resource allocation at job granularity rather than finer-grained container or function levels, leaving opportunities for more precise resource provisioning.

Future research directions appear promising across multiple fronts. Incorporating explicit workload phase detection before prediction could improve accuracy during startup and completion periods where current performance lags. Extending the framework to distributed multi-cluster scenarios introduces challenges around global coordination and network-aware placement that our single-cluster design does not address. Investigating self-supervised learning approaches might reduce dependence on labeled historical data, enabling deployment in environments with limited observability. The integration of cost models considering cloud pricing dynamics, particularly spot instance opportunities and reserved capacity constraints, would enhance practical applicability. Finally, exploring federated learning techniques could enable collaborative model training across organizations while preserving proprietary workload information, accelerating adoption through shared knowledge without compromising competitive advantages. As AI workloads continue proliferating and computing infrastructures grow increasingly heterogeneous, intelligent prediction-driven resource management systems will become indispensable for sustainable, efficient datacenter operations.

## Supplementary Information

Below is the link to the electronic supplementary material.

Supplementary Material 1

## Data Availability

The experimental results and analytical data supporting the findings of this study are provided in the Supplementary Materials accompanying this manuscript. The public datasets (Google Cluster Trace 2019 and Alibaba Cluster Trace 2020) are available from their respective repositories. Implementation code, trained model checkpoints, and detailed experimental configurations are available from the corresponding author, Peiqing Ye (15061073566@163.com), upon reasonable request. The Supplementary Materials include: (1) complete hyperparameter configurations for all baseline methods, (2) additional ablation study results, (3) per-dataset performance breakdowns, and (4) runtime profiling data across different cluster scales.

## Abbreviations

AI: Artificial intelligence
ARIMA: Autoregressive integrated moving average
CPU: Central processing unit
CV: Computer vision; coefficient of variation
FPGA: Field-programmable gate array
GPU: Graphics processing unit
GRU: Gated recurrent unit
LLM: Large Language model
LSTM: Long short-term memory
MAE: Mean absolute error
MAPE: Mean absolute percentage error
NAS: Neural architecture search
PPO: Proximal policy optimization
RNN: Recurrent neural network
RMSE: Root mean squared error
SLA: Service level agreement
TPU: Tensor processing unit
