# Supplementary material for: Attention-based workload prediction and dynamic resource allocation for heterogeneous computing environments
Source: Sci Rep. 2026 Feb 12;16:8571. doi: 10.1038/s41598-026-38622-4 (PMC12976089; doi:10.1038/s41598-026-38622-4)
Supplement: Supplementary file 1 — Supplementary Material 1 [file 41598_2026_38622_MOESM1_ESM.docx]

# Supplementary Materials

## Attention-Based Workload Prediction and Dynamic Resource Allocation for Heterogeneous Computing Environments

## Table S1. Detailed Hardware Specifications of Experimental Testbed

| Component | Specification | Quantity | Role |
| --- | --- | --- | --- |
| CPU Node Type A | Intel Xeon Gold 6248R (2×24 cores, 3.0GHz) | 8 | General-purpose computing |
| Memory (Type A) | DDR4-2933 ECC | 256GB/node | System memory |
| GPU Node Type B | NVIDIA V100 (32GB HBM2, 5120 CUDA cores) | 6 | Deep learning training |
| GPU Node Type C | NVIDIA A100 (80GB HBM2e, 6912 CUDA cores) | 4 | Large model training |
| TPU Node | Google TPU v3 (8 cores per pod) | 2 | Tensor operations |
| Network Switch | Mellanox SN3700 (100GbE) | 4 | Inter-node communication |
| Storage | NVMe SSD RAID-10 | 200TB total | Data storage |
| Network Latency | < 1μs (intra-rack), < 5μs (inter-rack) | - | Communication |

## Table S2. Hyperparameter Sensitivity Analysis for Attention Model

| Hyperparameter | Range Tested | Optimal Value | MAE Variation (%) | Training Time Impact |
| --- | --- | --- | --- | --- |
| Number of attention heads | {2, 4, 8, 16, 32} | 8 | ±3.2 | Linear increase |
| Embedding dimension | {128, 256, 512, 1024} | 512 | ±4.7 | Quadratic increase |
| Number of encoder layers | {2, 4, 6, 8, 12} | 6 | ±2.8 | Linear increase |
| Temporal window length | {32, 64, 128, 256, 512} | 128 | ±5.1 | Linear increase |
| Prediction horizon | {8, 16, 32, 64} | 32 | ±6.3 | Minimal |
| Dropout rate | {0.0, 0.1, 0.2, 0.3, 0.5} | 0.1 | ±2.1 | None |
| Learning rate | {1e-5, 5e-5, 1e-4, 5e-4, 1e-3} | 1e-4 | ±8.4 | Convergence speed |
| Batch size | {16, 32, 64, 128, 256} | 64 | ±1.9 | Memory usage |
| Warmup steps | {1000, 2000, 4000, 8000} | 4000 | ±1.5 | Early training stability |

## Table S3. Detailed Prediction Performance by Resource Type

| Method | Resource Type | MAE (%) | RMSE (%) | MAPE (%) | R² Score | 95% CI Coverage |
| --- | --- | --- | --- | --- | --- | --- |
| **Proposed** | CPU | 7.21 | 11.03 | 13.42 | 0.842 | 93.7% |
| **Proposed** | Memory | 8.15 | 12.89 | 15.23 | 0.818 | 91.2% |
| **Proposed** | GPU | 5.67 | 8.94 | 10.18 | 0.891 | 95.1% |
| **Proposed** | Network I/O | 9.34 | 14.21 | 17.56 | 0.789 | 89.4% |
| LSTM | CPU | 10.12 | 15.34 | 18.21 | 0.756 | 85.3% |
| LSTM | Memory | 11.87 | 17.62 | 21.34 | 0.712 | 82.1% |
| LSTM | GPU | 8.34 | 12.78 | 14.67 | 0.798 | 87.6% |
| LSTM | Network I/O | 12.45 | 18.93 | 23.12 | 0.683 | 79.8% |
| GRU | CPU | 9.45 | 14.23 | 16.89 | 0.778 | 86.9% |
| GRU | Memory | 10.98 | 16.45 | 19.87 | 0.734 | 84.2% |
| GRU | GPU | 7.89 | 11.94 | 13.56 | 0.812 | 88.9% |
| GRU | Network I/O | 11.67 | 17.56 | 21.45 | 0.701 | 81.5% |

## Table S4. Prediction Performance Across Different Forecast Horizons

| Method | Horizon (min) | MAE (%) | RMSE (%) | MAPE (%) | Degradation Rate |
| --- | --- | --- | --- | --- | --- |
| **Proposed** | 5 | 4.23 | 6.12 | 7.45 | Baseline |
| **Proposed** | 10 | 5.67 | 8.23 | 9.78 | +31.3% |
| **Proposed** | 15 | 6.84 | 10.52 | 12.15 | +63.1% |
| **Proposed** | 30 | 8.92 | 13.45 | 15.67 | +110.4% |
| **Proposed** | 60 | 12.34 | 18.67 | 21.23 | +185.0% |
| LSTM | 5 | 6.12 | 9.23 | 11.34 | Baseline |
| LSTM | 10 | 8.45 | 12.67 | 15.23 | +34.3% |
| LSTM | 15 | 10.23 | 15.34 | 18.45 | +62.7% |
| LSTM | 30 | 14.56 | 21.78 | 25.89 | +128.4% |
| LSTM | 60 | 21.34 | 31.45 | 37.23 | +228.4% |
| GRU | 5 | 5.78 | 8.67 | 10.56 | Baseline |
| GRU | 10 | 7.89 | 11.78 | 14.12 | +33.7% |
| GRU | 15 | 9.56 | 14.34 | 17.23 | +63.1% |
| GRU | 30 | 13.45 | 20.12 | 24.12 | +128.4% |
| GRU | 60 | 19.78 | 29.34 | 34.56 | +227.2% |

## Table S5. Workload Phase-Specific Prediction Performance

| Workload Phase | Duration (%) | Proposed MAE (%) | LSTM MAE (%) | GRU MAE (%) | Improvement |
| --- | --- | --- | --- | --- | --- |
| Job Startup | 8.3 | 14.82 | 22.45 | 20.34 | 34.0% |
| Data Loading | 12.7 | 11.23 | 18.67 | 16.89 | 39.9% |
| Steady Execution | 58.4 | 5.21 | 8.34 | 7.56 | 37.5% |
| Checkpoint/Sync | 6.2 | 9.87 | 15.23 | 14.12 | 35.2% |
| Resource Scaling | 4.8 | 12.45 | 19.78 | 17.89 | 37.1% |
| Job Completion | 9.6 | 18.34 | 26.78 | 24.56 | 31.5% |
| **Overall** | 100.0 | 6.84 | 10.52 | 9.56 | 35.0% |

## Table S6. Multi-Objective Optimization Weight Configuration Analysis

| Configuration | α (Completion Time) | β (Energy) | γ (Utilization) | Avg. Completion (min) | Energy (kWh) | Utilization (%) | SLA Violations (%) |
| --- | --- | --- | --- | --- | --- | --- | --- |
| Performance-First | 0.6 | 0.2 | 0.2 | 29.8 | 251.3 | 74.2 | 1.9 |
| Energy-First | 0.2 | 0.6 | 0.2 | 35.4 | 228.7 | 71.8 | 3.4 |
| Utilization-First | 0.2 | 0.2 | 0.6 | 33.7 | 243.5 | 81.2 | 3.1 |
| **Balanced** | 0.33 | 0.33 | 0.34 | 31.5 | 235.1 | 78.4 | 2.3 |
| Custom-A | 0.5 | 0.3 | 0.2 | 30.2 | 238.9 | 75.6 | 2.1 |
| Custom-B | 0.4 | 0.4 | 0.2 | 31.8 | 232.4 | 73.9 | 2.5 |

## Table S7. Resource Allocation Strategy Comparison Under Different Workload Intensities

| Workload Intensity | Strategy | Utilization (%) | Completion Time (min) | Energy (kWh) | SLA Violations (%) |
| --- | --- | --- | --- | --- | --- |
| Low (30% capacity) | First-Fit | 42.3 | 28.4 | 189.3 | 2.1 |
| Low | Best-Fit | 51.2 | 26.7 | 178.5 | 1.8 |
| Low | Kubernetes | 47.8 | 27.3 | 182.4 | 2.0 |
| Low | **Proposed** | 58.9 | 24.1 | 168.2 | 1.2 |
| Medium (60% capacity) | First-Fit | 58.7 | 42.3 | 284.5 | 8.4 |
| Medium | Best-Fit | 67.3 | 38.6 | 267.3 | 5.2 |
| Medium | Kubernetes | 62.1 | 40.1 | 276.8 | 6.7 |
| Medium | **Proposed** | 78.4 | 31.5 | 235.1 | 2.3 |
| High (90% capacity) | First-Fit | 71.2 | 58.9 | 412.7 | 18.3 |
| High | Best-Fit | 78.4 | 52.3 | 378.4 | 12.1 |
| High | Kubernetes | 74.6 | 55.6 | 398.2 | 14.7 |
| High | **Proposed** | 89.2 | 41.7 | 321.5 | 5.8 |

## Table S8. Ablation Study Results

| Configuration | MAE (%) | RMSE (%) | Utilization (%) | SLA Violations (%) | Energy (kWh) |
| --- | --- | --- | --- | --- | --- |
| Full Framework | 6.84 | 10.52 | 78.4 | 2.3 | 235.1 |
| w/o Spatial Attention | 9.23 | 14.12 | 72.1 | 4.1 | 251.8 |
| w/o Temporal Attention | 11.45 | 17.23 | 68.7 | 5.8 | 267.3 |
| w/o Multi-Head (Single Head) | 8.12 | 12.45 | 74.3 | 3.2 | 243.6 |
| w/o Positional Encoding | 7.89 | 11.98 | 75.8 | 2.9 | 239.4 |
| w/o Residual Connections | 8.56 | 13.12 | 73.2 | 3.5 | 248.7 |
| w/o Online Update | 9.78 (week 2) | 14.89 | 71.4 | 4.7 | 256.2 |
| w/o Preemption Mechanism | 6.84 | 10.52 | 76.9 | 6.9 | 241.3 |
| w/o Migration Mechanism | 6.84 | 10.52 | 74.2 | 4.2 | 247.8 |
| w/o Quantile Loss | 7.21 | 10.89 | 75.6 | 3.8 | 242.5 |

## Table S9. Computational Overhead Analysis

| Component | Time Complexity | Space Complexity | Avg. Latency (ms) | Memory Usage (GB) |
| --- | --- | --- | --- | --- |
| Temporal Attention | O(T² × D) | O(T × D) | 12.3 | 1.2 |
| Spatial Attention | O(W² × D) | O(W × D) | 8.7 | 0.8 |
| Multi-Head Computation | O(h × T² × D/h) | O(h × T × D/h) | 45.6 | 2.4 |
| Feedforward Network | O(T × D × D_ff) | O(D × D_ff) | 18.4 | 1.6 |
| Full Prediction Model | O(L × T² × D × h) | O(L × D² × h) | 98.7 | 4.8 |
| Workload Clustering | O(W × k × I) | O(W + k) | 23.4 | 0.3 |
| Allocation Optimization | O(W × N × k) | O(W + N) | 67.2 | 0.5 |
| Migration Decision | O(W × N) | O(W) | 12.8 | 0.1 |
| **Total Framework** | O(L×T²×D×h + W×N×k) | O(L×D²×h + W + N) | 189.3 | 5.6 |

*Note: T=sequence length, D=embedding dimension, h=attention heads, W=workloads, N=nodes, k=clusters, L=layers, I=iterations, D_ff=feedforward dimension*

## Table S10. Burst Workload Handling Performance

| Burst Scenario | Burst Magnitude | Detection Time (s) | Recovery Time (min) | SLA Maintained (%) | Resource Reallocation (%) |
| --- | --- | --- | --- | --- | --- |
| Single Job Spike | 2× baseline | 3.2 | 1.4 | 98.7 | 12.3 |
| Multi-Job Burst | 2× baseline | 4.8 | 2.1 | 97.8 | 23.5 |
| Cluster-Wide Surge | 3× baseline | 6.1 | 2.8 | 97.3 | 34.2 |
| GPU Memory Spike | 2.5× baseline | 4.2 | 1.9 | 98.1 | 18.7 |
| Network Congestion | 3× baseline | 5.7 | 2.5 | 96.9 | 28.4 |
| Cascading Failure | Variable | 8.3 | 4.2 | 94.5 | 45.6 |
| **Average** | 2.5× baseline | 5.4 | 2.5 | 97.2 | 27.1 |

## Table S11. Energy Efficiency Metrics by Hardware Type

| Hardware Type | Idle Power (W) | Peak Power (W) | Avg. Utilization (%) | Energy per Job (kWh) | PUE Contribution |
| --- | --- | --- | --- | --- | --- |
| CPU Node (Xeon Gold) | 85 | 180 | 72.3 | 0.42 | 1.12 |
| GPU Node (V100) | 45 | 300 | 78.5 | 0.89 | 1.08 |
| GPU Node (A100) | 55 | 400 | 82.1 | 1.12 | 1.06 |
| TPU Node (v3) | 120 | 450 | 85.4 | 1.28 | 1.05 |
| Network Infrastructure | 180 | 320 | - | - | 1.15 |
| Cooling System | - | - | - | - | 1.35 |
| **Total System** | 2,840 | 8,750 | 78.4 | 0.78 (avg) | 1.42 |

## Table S12. Cross-Dataset Generalization Performance

| Training Dataset | Test Dataset | MAE (%) | RMSE (%) | MAPE (%) | Generalization Gap |
| --- | --- | --- | --- | --- | --- |
| Google Cluster | Google Cluster | 6.84 | 10.52 | 12.15 | 0% (baseline) |
| Google Cluster | Alibaba Cluster | 11.23 | 16.78 | 19.34 | +59.2% |
| Google Cluster | Academic Cluster | 13.45 | 19.87 | 23.12 | +90.3% |
| Alibaba Cluster | Alibaba Cluster | 8.59 | 12.43 | 14.27 | 0% (baseline) |
| Alibaba Cluster | Google Cluster | 9.78 | 14.56 | 16.89 | +18.3% |
| Alibaba Cluster | Academic Cluster | 12.34 | 18.23 | 21.45 | +50.3% |
| Combined Dataset | Google Cluster | 7.12 | 10.89 | 12.67 | - |
| Combined Dataset | Alibaba Cluster | 8.89 | 12.78 | 14.56 | - |
| Combined Dataset | Academic Cluster | 10.12 | 15.34 | 17.89 | - |

## Table S13. Online Learning Adaptation Performance Over Time

| Time Period | Initial MAE (%) | Final MAE (%) | Drift Detected | Update Frequency | Convergence Time (hr) |
| --- | --- | --- | --- | --- | --- |
| Week 1 | 6.84 | 6.92 | No | Daily | - |
| Week 2 | 6.92 | 7.45 | Minor | Daily | 2.3 |
| Week 3 | 7.45 | 7.23 | Yes | 2× Daily | 4.1 |
| Week 4 | 7.23 | 6.98 | Minor | Daily | 1.8 |
| Week 5 | 6.98 | 8.12 | Major | 3× Daily | 6.7 |
| Week 6 | 8.12 | 7.34 | Recovery | 2× Daily | 5.2 |
| **Average** | 6.84 | 7.17 | - | 1.5× Daily | 4.0 |

## Supplementary Code

### Code S1. Multi-Head Spatial-Temporal Attention Module Implementation

import torch
import torch.nn as nn
import torch.nn.functional as F
import math

class MultiHeadSpatialTemporalAttention(nn.Module):
 """
 Multi-Head Spatial-Temporal Attention for Workload Prediction
 Implements separate temporal and spatial attention mechanisms
 """

 def __init__(self, d_model=512, n_heads=8, d_ff=2048, dropout=0.1):
 super().__init__()
 self.d_model = d_model
 self.n_heads = n_heads
 self.d_k = d_model // n_heads

 # Temporal attention projections
 self.W_q_temp = nn.Linear(d_model, d_model)
 self.W_k_temp = nn.Linear(d_model, d_model)
 self.W_v_temp = nn.Linear(d_model, d_model)
 self.W_o_temp = nn.Linear(d_model, d_model)

 # Spatial attention projections
 self.W_q_spatial = nn.Linear(d_model, d_model)
 self.W_k_spatial = nn.Linear(d_model, d_model)
 self.W_v_spatial = nn.Linear(d_model, d_model)
 self.W_o_spatial = nn.Linear(d_model, d_model)

 # Feedforward network
 self.ffn = nn.Sequential(
 nn.Linear(d_model, d_ff),
 nn.GELU(),
 nn.Dropout(dropout),
 nn.Linear(d_ff, d_model)
 )

 # Layer normalization
 self.norm1 = nn.LayerNorm(d_model)
 self.norm2 = nn.LayerNorm(d_model)
 self.norm3 = nn.LayerNorm(d_model)

 self.dropout = nn.Dropout(dropout)

 def temporal_attention(self, x):
 """
 Compute temporal attention across time steps
 x: (batch, time, workloads, d_model)
 """
 B, T, W, D = x.shape

 # Reshape for temporal attention: treat each workload independently
 x_temp = x.permute(0, 2, 1, 3).reshape(B * W, T, D)

 # Project to Q, K, V
 Q = self.W_q_temp(x_temp).view(B * W, T, self.n_heads, self.d_k).transpose(1, 2)
 K = self.W_k_temp(x_temp).view(B * W, T, self.n_heads, self.d_k).transpose(1, 2)
 V = self.W_v_temp(x_temp).view(B * W, T, self.n_heads, self.d_k).transpose(1, 2)

 # Scaled dot-product attention
 scores = torch.matmul(Q, K.transpose(-2, -1)) / math.sqrt(self.d_k)
 attn_weights = F.softmax(scores, dim=-1)
 attn_weights = self.dropout(attn_weights)

 # Apply attention to values
 context = torch.matmul(attn_weights, V)
 context = context.transpose(1, 2).reshape(B * W, T, D)
 output = self.W_o_temp(context)

 # Reshape back
 output = output.view(B, W, T, D).permute(0, 2, 1, 3)

 return output, attn_weights

 def spatial_attention(self, x):
 """
 Compute spatial attention across workload types
 x: (batch, time, workloads, d_model)
 """
 B, T, W, D = x.shape

 # Reshape for spatial attention: treat each time step independently
 x_spatial = x.reshape(B * T, W, D)

 # Project to Q, K, V
 Q = self.W_q_spatial(x_spatial).view(B * T, W, self.n_heads, self.d_k).transpose(1, 2)
 K = self.W_k_spatial(x_spatial).view(B * T, W, self.n_heads, self.d_k).transpose(1, 2)
 V = self.W_v_spatial(x_spatial).view(B * T, W, self.n_heads, self.d_k).transpose(1, 2)

 # Scaled dot-product attention
 scores = torch.matmul(Q, K.transpose(-2, -1)) / math.sqrt(self.d_k)
 attn_weights = F.softmax(scores, dim=-1)
 attn_weights = self.dropout(attn_weights)

 # Apply attention to values
 context = torch.matmul(attn_weights, V)
 context = context.transpose(1, 2).reshape(B * T, W, D)
 output = self.W_o_spatial(context)

 # Reshape back
 output = output.view(B, T, W, D)

 return output, attn_weights

 def forward(self, x):
 """
 Forward pass with temporal attention followed by spatial attention
 x: (batch, time, workloads, d_model)
 """
 # Temporal attention with residual connection
 temp_out, temp_attn = self.temporal_attention(x)
 x = self.norm1(x + self.dropout(temp_out))

 # Spatial attention with residual connection
 spatial_out, spatial_attn = self.spatial_attention(x)
 x = self.norm2(x + self.dropout(spatial_out))

 # Feedforward network with residual connection
 ffn_out = self.ffn(x)
 x = self.norm3(x + self.dropout(ffn_out))

 return x, {'temporal': temp_attn, 'spatial': spatial_attn}


class PositionalEncoding(nn.Module):
 """Sinusoidal positional encoding for temporal sequences"""

 def __init__(self, d_model, max_len=5000):
 super().__init__()

 pe = torch.zeros(max_len, d_model)
 position = torch.arange(0, max_len, dtype=torch.float).unsqueeze(1)
 div_term = torch.exp(torch.arange(0, d_model, 2).float() * (-math.log(10000.0) / d_model))

 pe[:, 0::2] = torch.sin(position * div_term)
 pe[:, 1::2] = torch.cos(position * div_term)
 pe = pe.unsqueeze(0)

 self.register_buffer('pe', pe)

 def forward(self, x):
 """x: (batch, time, d_model)"""
 return x + self.pe[:, :x.size(1), :]


class WorkloadPredictionModel(nn.Module):
 """
 Complete workload prediction model with spatial-temporal attention
 """

 def __init__(self, input_dim=4, d_model=512, n_heads=8, n_layers=6,
 d_ff=2048, dropout=0.1, pred_horizon=32, n_workloads=10):
 super().__init__()

 self.input_dim = input_dim
 self.d_model = d_model
 self.pred_horizon = pred_horizon
 self.n_workloads = n_workloads

 # Input embedding
 self.input_embedding = nn.Linear(input_dim, d_model)

 # Positional encoding
 self.pos_encoding = PositionalEncoding(d_model)

 # Stack of spatial-temporal attention layers
 self.attention_layers = nn.ModuleList([
 MultiHeadSpatialTemporalAttention(d_model, n_heads, d_ff, dropout)
 for _ in range(n_layers)
 ])

 # Output projection for point prediction
 self.output_proj = nn.Linear(d_model, input_dim)

 # Quantile prediction heads (for uncertainty estimation)
 self.quantile_heads = nn.ModuleDict({
 'q10': nn.Linear(d_model, input_dim),
 'q50': nn.Linear(d_model, input_dim),
 'q90': nn.Linear(d_model, input_dim)
 })

 # Prediction horizon projection
 self.horizon_proj = nn.Linear(d_model, d_model * pred_horizon)

 self.dropout = nn.Dropout(dropout)

 def forward(self, x, return_attention=False):
 """
 x: (batch, time, workloads, input_dim)
 Returns predictions for next pred_horizon steps
 """
 B, T, W, _ = x.shape

 # Input embedding
 x = self.input_embedding(x) # (B, T, W, d_model)

 # Add positional encoding (applied along time dimension)
 x_reshaped = x.view(B * W, T, self.d_model)
 x_reshaped = self.pos_encoding(x_reshaped)
 x = x_reshaped.view(B, T, W, self.d_model)

 x = self.dropout(x)

 # Apply attention layers
 attention_weights = []
 for layer in self.attention_layers:
 x, attn = layer(x)
 attention_weights.append(attn)

 # Use last time step for prediction
 x_last = x[:, -1, :, :] # (B, W, d_model)

 # Project to prediction horizon
 x_horizon = self.horizon_proj(x_last) # (B, W, d_model * pred_horizon)
 x_horizon = x_horizon.view(B, W, self.pred_horizon, self.d_model)
 x_horizon = x_horizon.permute(0, 2, 1, 3) # (B, pred_horizon, W, d_model)

 # Point prediction
 point_pred = self.output_proj(x_horizon) # (B, pred_horizon, W, input_dim)

 # Quantile predictions
 quantile_preds = {
 q: head(x_horizon) for q, head in self.quantile_heads.items()
 }

 outputs = {
 'point': point_pred,
 'quantiles': quantile_preds
 }

 if return_attention:
 outputs['attention'] = attention_weights

 return outputs


class QuantileLoss(nn.Module):
 """Combined MSE and quantile loss for prediction with uncertainty"""

 def __init__(self, quantiles=[0.1, 0.5, 0.9], mse_weight=1.0, quantile_weight=0.5):
 super().__init__()
 self.quantiles = quantiles
 self.mse_weight = mse_weight
 self.quantile_weight = quantile_weight

 def forward(self, predictions, targets, capacity_constraints=None):
 """
 predictions: dict with 'point' and 'quantiles'
 targets: (batch, horizon, workloads, features)
 capacity_constraints: optional tensor for constraint violation penalty
 """
 point_pred = predictions['point']
 quantile_preds = predictions['quantiles']

 # MSE loss for point prediction
 mse_loss = F.mse_loss(point_pred, targets)

 # Quantile losses
 quantile_losses = []
 for q, q_name in zip(self.quantiles, ['q10', 'q50', 'q90']):
 q_pred = quantile_preds[q_name]
 errors = targets - q_pred
 quantile_loss = torch.max(q * errors, (q - 1) * errors).mean()
 quantile_losses.append(quantile_loss)

 total_quantile_loss = sum(quantile_losses)

 # Capacity constraint penalty (optional)
 constraint_loss = 0.0
 if capacity_constraints is not None:
 violations = F.relu(point_pred - capacity_constraints)
 constraint_loss = violations.mean()

 total_loss = (self.mse_weight * mse_loss +
 self.quantile_weight * total_quantile_loss +
 0.1 * constraint_loss)

 return total_loss, {
 'mse': mse_loss.item(),
 'quantile': total_quantile_loss.item(),
 'constraint': constraint_loss if isinstance(constraint_loss, float) else constraint_loss.item()
 }

### Code S2. Dynamic Resource Allocation Algorithm

import numpy as np
from dataclasses import dataclass
from typing import List, Dict, Tuple, Optional
from enum import Enum
import heapq

class ResourceType(Enum):
 CPU = "cpu"
 MEMORY = "memory"
 GPU = "gpu"
 NETWORK = "network"

@dataclass
class ComputeNode:
 """Representation of a heterogeneous compute node"""
 node_id: str
 node_type: str # 'cpu', 'gpu_v100', 'gpu_a100', 'tpu'
 cpu_cores: int
 memory_gb: float
 gpu_memory_gb: float
 power_consumption_w: float

 # Current state
 available_cpu: int = None
 available_memory: float = None
 available_gpu: float = None
 current_workloads: List = None

 def __post_init__(self):
 if self.available_cpu is None:
 self.available_cpu = self.cpu_cores
 if self.available_memory is None:
 self.available_memory = self.memory_gb
 if self.available_gpu is None:
 self.available_gpu = self.gpu_memory_gb
 if self.current_workloads is None:
 self.current_workloads = []

 def utilization(self) -> float:
 """Calculate overall node utilization"""
 cpu_util = 1 - (self.available_cpu / self.cpu_cores)
 mem_util = 1 - (self.available_memory / self.memory_gb)
 gpu_util = 1 - (self.available_gpu / self.gpu_memory_gb) if self.gpu_memory_gb > 0 else 0

 weights = [0.3, 0.3, 0.4] if self.gpu_memory_gb > 0 else [0.5, 0.5, 0]
 return weights[0] * cpu_util + weights[1] * mem_util + weights[2] * gpu_util


@dataclass
class Workload:
 """Representation of a workload/job"""
 workload_id: str
 workload_type: str # 'training', 'inference', 'batch'
 priority: int # Higher is more important

 # Resource requirements
 cpu_required: int
 memory_required: float
 gpu_required: float

 # Predicted requirements (from attention model)
 predicted_cpu: np.ndarray = None # Time series prediction
 predicted_memory: np.ndarray = None
 predicted_gpu: np.ndarray = None
 prediction_confidence: float = 0.5

 # Runtime state
 assigned_node: str = None
 start_time: float = None
 estimated_completion: float = None


class DynamicResourceAllocator:
 """
 Dynamic resource allocation framework with prediction-driven scheduling
 """

 def __init__(self, nodes: List[ComputeNode],
 alpha: float = 0.33, # Completion time weight
 beta: float = 0.33, # Energy weight
 gamma: float = 0.34, # Utilization weight
 preemption_threshold: float = 0.9,
 migration_gain_threshold: float = 0.1):

 self.nodes = {n.node_id: n for n in nodes}
 self.alpha = alpha
 self.beta = beta
 self.gamma = gamma
 self.preemption_threshold = preemption_threshold
 self.migration_gain_threshold = migration_gain_threshold

 # Workload-to-node type affinity scores
 self.affinity_matrix = {
 ('training', 'gpu_a100'): 1.0,
 ('training', 'gpu_v100'): 0.8,
 ('training', 'tpu'): 0.9,
 ('training', 'cpu'): 0.3,
 ('inference', 'gpu_a100'): 0.9,
 ('inference', 'gpu_v100'): 0.85,
 ('inference', 'tpu'): 0.7,
 ('inference', 'cpu'): 0.5,
 ('batch', 'cpu'): 0.9,
 ('batch', 'gpu_v100'): 0.6,
 ('batch', 'gpu_a100'): 0.5,
 ('batch', 'tpu'): 0.4,
 }

 def cluster_workloads(self, workloads: List[Workload], k: int = 4) -> Dict[int, List[Workload]]:
 """
 Cluster workloads by resource profile similarity using k-means
 """
 if len(workloads) <= k:
 return {i: [w] for i, w in enumerate(workloads)}

 # Extract feature vectors
 features = np.array([
 [w.cpu_required, w.memory_required, w.gpu_required]
 for w in workloads
 ])

 # Normalize features
 features_norm = (features - features.mean(axis=0)) / (features.std(axis=0) + 1e-8)

 # Simple k-means implementation
 centroids = features_norm[np.random.choice(len(features_norm), k, replace=False)]

 for _ in range(10): # Max iterations
 # Assign to nearest centroid
 distances = np.linalg.norm(features_norm[:, np.newaxis] - centroids, axis=2)
 assignments = np.argmin(distances, axis=1)

 # Update centroids
 new_centroids = np.array([
 features_norm[assignments == i].mean(axis=0) if np.sum(assignments == i) > 0
 else centroids[i]
 for i in range(k)
 ])

 if np.allclose(centroids, new_centroids):
 break
 centroids = new_centroids

 # Group workloads by cluster
 clusters = {i: [] for i in range(k)}
 for w, c in zip(workloads, assignments):
 clusters[c].append(w)

 return clusters

 def compute_allocation_utility(self, workload: Workload, node: ComputeNode) -> float:
 """
 Compute utility of assigning workload to node
 Equation (18) in the paper
 """
 # Check feasibility
 if (workload.cpu_required > node.available_cpu or
 workload.memory_required > node.available_memory or
 workload.gpu_required > node.available_gpu):
 return float('-inf')

 # Affinity score
 affinity = self.affinity_matrix.get(
 (workload.workload_type, node.node_type), 0.5
 )

 # Utilization improvement
 current_util = node.utilization()

 # Simulate allocation
 temp_cpu = node.available_cpu - workload.cpu_required
 temp_mem = node.available_memory - workload.memory_required
 temp_gpu = node.available_gpu - workload.gpu_required

 new_util = 1 - (temp_cpu / node.cpu_cores) * 0.3 - \
 (temp_mem / node.memory_gb) * 0.3 - \
 (temp_gpu / node.gpu_memory_gb if node.gpu_memory_gb > 0 else 0) * 0.4

 delta_util = new_util - current_util

 # Energy cost (normalized)
 energy_cost = node.power_consumption_w / 500.0 # Normalize by reference power

 # Estimated execution time improvement based on affinity
 time_factor = 1.0 / (affinity + 0.1)

 # Combined utility (Equation 18)
 utility = (self.gamma * delta_util -
 self.beta * energy_cost * time_factor +
 self.alpha * affinity)

 return utility

 def allocate_workloads(self, workloads: List[Workload],
 predictions: Dict[str, np.ndarray] = None) -> Dict[str, str]:
 """
 Main allocation algorithm
 Returns mapping of workload_id -> node_id
 """
 allocation = {}

 # Update workload predictions if provided
 if predictions:
 for w in workloads:
 if w.workload_id in predictions:
 pred = predictions[w.workload_id]
 w.predicted_cpu = pred.get('cpu')
 w.predicted_memory = pred.get('memory')
 w.predicted_gpu = pred.get('gpu')
 w.prediction_confidence = pred.get('confidence', 0.5)

 # Cluster workloads
 clusters = self.cluster_workloads(workloads)

 # Process each cluster
 for cluster_id, cluster_workloads in clusters.items():
 # Sort by priority (descending)
 cluster_workloads.sort(key=lambda w: w.priority, reverse=True)

 for workload in cluster_workloads:
 # Adjust requirements based on prediction confidence
 safety_margin = 1 + (1 - workload.prediction_confidence) * 0.2
 adjusted_cpu = workload.cpu_required * safety_margin
 adjusted_mem = workload.memory_required * safety_margin
 adjusted_gpu = workload.gpu_required * safety_margin

 # Find best node
 best_node = None
 best_utility = float('-inf')

 for node_id, node in self.nodes.items():
 utility = self.compute_allocation_utility(workload, node)
 if utility > best_utility:
 best_utility = utility
 best_node = node_id

 if best_node is not None:
 # Perform allocation
 node = self.nodes[best_node]
 node.available_cpu -= workload.cpu_required
 node.available_memory -= workload.memory_required
 node.available_gpu -= workload.gpu_required
 node.current_workloads.append(workload.workload_id)

 workload.assigned_node = best_node
 allocation[workload.workload_id] = best_node
 else:
 # Trigger preemption if needed
 preempted = self.try_preemption(workload)
 if preempted:
 allocation[workload.workload_id] = workload.assigned_node

 return allocation

 def try_preemption(self, high_priority_workload: Workload) -> bool:
 """
 Attempt to preempt lower priority workloads (Equation 14)
 """
 for node_id, node in self.nodes.items():
 if node.utilization() < self.preemption_threshold:
 continue

 # Find preemptable workloads
 preemptable = []
 for w_id in node.current_workloads:
 # In real implementation, would look up workload priority
 # Here we simulate with placeholder
 preemptable.append(w_id)

 # If we can free enough resources by preemption
 if preemptable:
 # Preempt lowest priority workload
 # In practice, would checkpoint and migrate
 preempt_id = preemptable[0]
 node.current_workloads.remove(preempt_id)

 # Try allocation again
 utility = self.compute_allocation_utility(high_priority_workload, node)
 if utility > float('-inf'):
 node.available_cpu -= high_priority_workload.cpu_required
 node.available_memory -= high_priority_workload.memory_required
 node.available_gpu -= high_priority_workload.gpu_required
 node.current_workloads.append(high_priority_workload.workload_id)
 high_priority_workload.assigned_node = node_id
 return True

 return False

 def compute_migration_gain(self, workload: Workload,
 source_node: ComputeNode,
 target_node: ComputeNode,
 transfer_cost: float = 0.1) -> float:
 """
 Compute migration gain (Equation 15)
 """
 # Estimate time improvement
 source_affinity = self.affinity_matrix.get(
 (workload.workload_type, source_node.node_type), 0.5
 )
 target_affinity = self.affinity_matrix.get(
 (workload.workload_type, target_node.node_type), 0.5
 )

 time_improvement = (source_affinity - target_affinity) / source_affinity

 # Migration cost includes checkpoint, transfer, warmup
 migration_overhead = transfer_cost * (
 workload.memory_required / 10.0 # Proportional to memory
 )

 gain = time_improvement * workload.priority - migration_overhead
 return gain

 def evaluate_migrations(self, workloads: List[Workload]) -> List[Tuple[str, str, str]]:
 """
 Evaluate and recommend beneficial migrations
 Returns list of (workload_id, source_node, target_node)
 """
 migrations = []

 for workload in workloads:
 if workload.assigned_node is None:
 continue

 source_node = self.nodes[workload.assigned_node]

 for target_id, target_node in self.nodes.items():
 if target_id == workload.assigned_node:
 continue

 # Check if target has capacity
 if (workload.cpu_required <= target_node.available_cpu and
 workload.memory_required <= target_node.available_memory and
 workload.gpu_required <= target_node.available_gpu):

 gain = self.compute_migration_gain(
 workload, source_node, target_node
 )

 if gain > self.migration_gain_threshold:
 migrations.append((
 workload.workload_id,
 workload.assigned_node,
 target_id,
 gain
 ))

 # Sort by gain and return top migrations
 migrations.sort(key=lambda x: x[3], reverse=True)
 return [(m[0], m[1], m[2]) for m in migrations[:5]]

 def get_system_metrics(self) -> Dict:
 """Calculate system-wide metrics"""
 total_cpu = sum(n.cpu_cores for n in self.nodes.values())
 total_memory = sum(n.memory_gb for n in self.nodes.values())
 total_gpu = sum(n.gpu_memory_gb for n in self.nodes.values())

 used_cpu = sum(n.cpu_cores - n.available_cpu for n in self.nodes.values())
 used_memory = sum(n.memory_gb - n.available_memory for n in self.nodes.values())
 used_gpu = sum(n.gpu_memory_gb - n.available_gpu for n in self.nodes.values())

 total_power = sum(
 n.power_consumption_w * n.utilization()
 for n in self.nodes.values()
 )

 return {
 'cpu_utilization': used_cpu / total_cpu,
 'memory_utilization': used_memory / total_memory,
 'gpu_utilization': used_gpu / total_gpu if total_gpu > 0 else 0,
 'overall_utilization': np.mean([
 used_cpu / total_cpu,
 used_memory / total_memory,
 used_gpu / total_gpu if total_gpu > 0 else 0
 ]),
 'total_power_consumption': total_power,
 'active_nodes': sum(1 for n in self.nodes.values() if n.utilization() > 0.01),
 'total_nodes': len(self.nodes)
 }

### Code S3. Training Loop and Online Update

import torch
import torch.optim as optim
from torch.utils.data import DataLoader, Dataset
import numpy as np
from typing import Dict, List, Tuple
import logging

logging.basicConfig(level=logging.INFO)
logger = logging.getLogger(__name__)


class WorkloadDataset(Dataset):
 """Dataset for workload time series"""

 def __init__(self, data: np.ndarray, window_size: int = 128,
 horizon: int = 32, n_workloads: int = 10):
 """
 data: (time_steps, workloads, features)
 """
 self.data = torch.FloatTensor(data)
 self.window_size = window_size
 self.horizon = horizon
 self.n_workloads = n_workloads

 self.valid_indices = list(range(
 window_size,
 len(data) - horizon
 ))

 def __len__(self):
 return len(self.valid_indices)

 def __getitem__(self, idx):
 t = self.valid_indices[idx]

 x = self.data[t - self.window_size:t] # (window, workloads, features)
 y = self.data[t:t + self.horizon] # (horizon, workloads, features)

 return x, y


class WorkloadPredictor:
 """
 Complete prediction system with training and online update capabilities
 """

 def __init__(self, model: 'WorkloadPredictionModel',
 device: str = 'cuda',
 learning_rate: float = 1e-4,
 weight_decay: float = 0.01):

 self.model = model.to(device)
 self.device = device

 self.optimizer = optim.AdamW(
 model.parameters(),
 lr=learning_rate,
 weight_decay=weight_decay
 )

 self.scheduler = optim.lr_scheduler.CosineAnnealingWarmRestarts(
 self.optimizer, T_0=10, T_mult=2
 )

 self.loss_fn = QuantileLoss(
 quantiles=[0.1, 0.5, 0.9],
 mse_weight=1.0,
 quantile_weight=0.5
 )

 self.training_history = []
 self.online_buffer = []
 self.buffer_size = 1000

 def train_epoch(self, dataloader: DataLoader) -> Dict[str, float]:
 """Train for one epoch"""
 self.model.train()
 total_loss = 0
 total_mse = 0
 total_quantile = 0
 n_batches = 0

 for batch_x, batch_y in dataloader:
 batch_x = batch_x.to(self.device)
 batch_y = batch_y.to(self.device)

 self.optimizer.zero_grad()

 predictions = self.model(batch_x)
 loss, loss_components = self.loss_fn(predictions, batch_y)

 loss.backward()

 # Gradient clipping
 torch.nn.utils.clip_grad_norm_(self.model.parameters(), max_norm=1.0)

 self.optimizer.step()

 total_loss += loss.item()
 total_mse += loss_components['mse']
 total_quantile += loss_components['quantile']
 n_batches += 1

 self.scheduler.step()

 return {
 'loss': total_loss / n_batches,
 'mse': total_mse / n_batches,
 'quantile': total_quantile / n_batches
 }

 def evaluate(self, dataloader: DataLoader) -> Dict[str, float]:
 """Evaluate model on validation/test data"""
 self.model.eval()

 all_predictions = []
 all_targets = []

 with torch.no_grad():
 for batch_x, batch_y in dataloader:
 batch_x = batch_x.to(self.device)
 batch_y = batch_y.to(self.device)

 predictions = self.model(batch_x)

 all_predictions.append(predictions['point'].cpu().numpy())
 all_targets.append(batch_y.cpu().numpy())

 predictions = np.concatenate(all_predictions, axis=0)
 targets = np.concatenate(all_targets, axis=0)

 # Compute metrics
 mae = np.mean(np.abs(predictions - targets))
 rmse = np.sqrt(np.mean((predictions - targets) ** 2))
 mape = np.mean(np.abs((predictions - targets) / (targets + 1e-8))) * 100

 # R² score
 ss_res = np.sum((targets - predictions) ** 2)
 ss_tot = np.sum((targets - targets.mean()) ** 2)
 r2 = 1 - (ss_res / ss_tot)

 return {
 'mae': mae,
 'rmse': rmse,
 'mape': mape,
 'r2': r2
 }

 def curriculum_train(self, train_data: np.ndarray, val_data: np.ndarray,
 epochs: int = 200, batch_size: int = 64,
 warmup_steps: int = 4000) -> List[Dict]:
 """
 Curriculum learning: gradually increase prediction horizon
 """
 history = []

 # Curriculum stages: progressively longer horizons
 horizons = [8, 16, 32]
 epochs_per_stage = epochs // len(horizons)

 for stage, horizon in enumerate(horizons):
 logger.info(f"Curriculum stage {stage + 1}: horizon = {horizon}")

 # Create datasets for current horizon
 train_dataset = WorkloadDataset(train_data, horizon=horizon)
 val_dataset = WorkloadDataset(val_data, horizon=horizon)

 train_loader = DataLoader(
 train_dataset, batch_size=batch_size, shuffle=True, num_workers=4
 )
 val_loader = DataLoader(
 val_dataset, batch_size=batch_size, shuffle=False, num_workers=4
 )

 for epoch in range(epochs_per_stage):
 train_metrics = self.train_epoch(train_loader)

 if (epoch + 1) % 10 == 0:
 val_metrics = self.evaluate(val_loader)

 logger.info(
 f"Stage {stage + 1}, Epoch {epoch + 1}: "
 f"Train Loss = {train_metrics['loss']:.4f}, "
 f"Val MAE = {val_metrics['mae']:.4f}, "
 f"Val MAPE = {val_metrics['mape']:.2f}%"
 )

 history.append({
 'stage': stage,
 'epoch': epoch,
 'horizon': horizon,
 **train_metrics,
 **{f'val_{k}': v for k, v in val_metrics.items()}
 })

 self.training_history = history
 return history

 def online_update(self, new_observations: np.ndarray,
 update_lr: float = 1e-5,
 max_updates: int = 10):
 """
 Online model update with new observations (Equation 19)
 """
 # Add to buffer
 self.online_buffer.extend(new_observations.tolist())

 # Keep buffer size manageable
 if len(self.online_buffer) > self.buffer_size:
 self.online_buffer = self.online_buffer[-self.buffer_size:]

 # Only update if we have enough data
 if len(self.online_buffer) < 200:
 return

 # Create mini-dataset from recent data
 buffer_array = np.array(self.online_buffer)

 # Adjust learning rate for stability
 for param_group in self.optimizer.param_groups:
 param_group['lr'] = update_lr

 self.model.train()

 dataset = WorkloadDataset(buffer_array, window_size=64, horizon=16)
 loader = DataLoader(dataset, batch_size=32, shuffle=True)

 for _ in range(max_updates):
 for batch_x, batch_y in loader:
 batch_x = batch_x.to(self.device)
 batch_y = batch_y.to(self.device)

 self.optimizer.zero_grad()
 predictions = self.model(batch_x)
 loss, _ = self.loss_fn(predictions, batch_y)
 loss.backward()

 # Conservative gradient clipping for online updates
 torch.nn.utils.clip_grad_norm_(self.model.parameters(), max_norm=0.5)
 self.optimizer.step()

 logger.info(f"Online update completed with {len(self.online_buffer)} samples")

 def predict(self, input_sequence: np.ndarray,
 return_uncertainty: bool = True) -> Dict[str, np.ndarray]:
 """
 Make predictions with uncertainty estimates
 """
 self.model.eval()

 with torch.no_grad():
 x = torch.FloatTensor(input_sequence).unsqueeze(0).to(self.device)
 outputs = self.model(x, return_attention=True)

 result = {
 'point': outputs['point'][0].cpu().numpy(),
 }

 if return_uncertainty:
 result['lower'] = outputs['quantiles']['q10'][0].cpu().numpy()
 result['upper'] = outputs['quantiles']['q90'][0].cpu().numpy()
 result['confidence'] = 1 - np.mean(
 result['upper'] - result['lower']
 ) / (np.mean(result['point']) + 1e-8)

 return result

 def save_checkpoint(self, path: str):
 """Save model checkpoint"""
 torch.save({
 'model_state_dict': self.model.state_dict(),
 'optimizer_state_dict': self.optimizer.state_dict(),
 'scheduler_state_dict': self.scheduler.state_dict(),
 'training_history': self.training_history,
 'online_buffer': self.online_buffer
 }, path)
 logger.info(f"Checkpoint saved to {path}")

 def load_checkpoint(self, path: str):
 """Load model checkpoint"""
 checkpoint = torch.load(path, map_location=self.device)
 self.model.load_state_dict(checkpoint['model_state_dict'])
 self.optimizer.load_state_dict(checkpoint['optimizer_state_dict'])
 self.scheduler.load_state_dict(checkpoint['scheduler_state_dict'])
 self.training_history = checkpoint.get('training_history', [])
 self.online_buffer = checkpoint.get('online_buffer', [])
 logger.info(f"Checkpoint loaded from {path}")


# Example usage
if __name__ == "__main__":
 # Initialize model
 model = WorkloadPredictionModel(
 input_dim=4,
 d_model=512,
 n_heads=8,
 n_layers=6,
 d_ff=2048,
 dropout=0.1,
 pred_horizon=32,
 n_workloads=10
 )

 # Initialize predictor
 predictor = WorkloadPredictor(
 model=model,
 device='cuda' if torch.cuda.is_available() else 'cpu',
 learning_rate=1e-4
 )

 # Generate synthetic data for demonstration
 np.random.seed(42)
 time_steps = 10000
 n_workloads = 10
 n_features = 4 # cpu, memory, gpu, network

 # Synthetic workload data with patterns
 t = np.linspace(0, 100 * np.pi, time_steps)
 base_pattern = np.sin(t)[:, np.newaxis, np.newaxis] * 0.3 + 0.5
 noise = np.random.randn(time_steps, n_workloads, n_features) * 0.1
 data = np.clip(base_pattern + noise, 0, 1).astype(np.float32)

 # Split data
 train_data = data[:7000]
 val_data = data[7000:8500]
 test_data = data[8500:]

 # Train with curriculum learning
 history = predictor.curriculum_train(
 train_data, val_data,
 epochs=60, # Reduced for demo
 batch_size=64
 )

 # Evaluate on test set
 test_dataset = WorkloadDataset(test_data, window_size=128, horizon=32)
 test_loader = DataLoader(test_dataset, batch_size=64, shuffle=False)

 test_metrics = predictor.evaluate(test_loader)
 print(f"\nTest Results:")
 print(f" MAE: {test_metrics['mae']:.4f}")
 print(f" RMSE: {test_metrics['rmse']:.4f}")
 print(f" MAPE: {test_metrics['mape']:.2f}%")
 print(f" R²: {test_metrics['r2']:.4f}")

## Table S14. Notation Summary

| Symbol | Description | Typical Value/Range |
| --- | --- | --- |
| T | Temporal window length | 128 time steps |
| D | Embedding dimension | 512 |
| h | Number of attention heads | 8 |
| d_k | Key/Query dimension per head | D/h = 64 |
| L | Number of encoder layers | 6 |
| H | Prediction horizon | 32 time steps |
| W | Number of concurrent workloads | 10-1000 |
| N | Number of computing nodes | 20 |
| \alpha | Completion time weight | 0.33 |
| \beta | Energy consumption weight | 0.33 |
| \gamma | Utilization weight | 0.34 |
| \theta_{high} | Preemption threshold | 0.9 |
| \eta | Learning rate | 10^{-4} |
| \tau | Online update interval | 1 hour |
| \lambda | Constraint violation penalty | 0.1 |
| Q, K, V | Query, Key, Value matrices | \mathbb{R}^{T \times D} |
